# Woven bone formation and mineralization by rat mesenchymal stromal cells imply increased expression of the intermediate filament desmin

**DOI:** 10.3389/fendo.2023.1234569

**Published:** 2023-09-04

**Authors:** Giusy Di Conza, Fulvio Barbaro, Nicoletta Zini, Giulia Spaletta, Giulia Remaggi, Lisa Elviri, Salvatore Mosca, Silvio Caravelli, Massimiliano Mosca, Roberto Toni

**Affiliations:** ^1^Department of Medicine and Surgery - DIMEC, Unit of Biomedical, Biotechnological and Translational Sciences (S.BI.BI.T.), Laboratory of Regenerative Morphology and Bioartificial Structures (Re.Mo.Bio.S.), and Museum and Historical Library of Biomedicine - BIOMED, University of Parma, Parma, Italy; ^2^Unit of Bologna, National Research Council of Italy (CNR) Institute of Molecular Genetics “Luigi Luca Cavalli-Sforza”, Bologna, Italy; ^3^IRCCS Istituto Ortopedico Rizzoli, Bologna, Italy; ^4^Department of Statistical Sciences, University of Bologna, Bologna, Italy; ^5^Food and Drug Department, University of Parma, Parma, Italy; ^6^Course on Disorders of the Locomotor System, Fellow Program in Orthopaedics and Traumatology, University Vita-Salute San Raffaele, Milan, Italy; ^7^II Clinic of Orthopedic and Traumatology, IRCCS Istituto Ortopedico Rizzoli, Bologna, Italy; ^8^Endocrinology, Diabetes, and Nutrition Disorders Outpatient Clinic, Osteoporosis, Nutrition, Endocrinology, and Innovative Therapies (OSTEONET) Unit, Galliera Medical Center (GMC), San Venanzio di Galliera, BO, Italy; ^9^Section IV - Medical Sciences, Academy of Sciences of the Institute of Bologna, Bologna, Italy; ^10^Department of Medicine, Division of Endocrinology, Diabetes, and Metabolism, Tufts Medical Center - Tufts University School of Medicine, Boston, MA, United States

**Keywords:** desmin, intermediate filaments, cytoskeleton, mesenchymal stromal cells, woven bone, osteogenesis, non-union fractures, metabolic skeletal dysplasia

## Abstract

**Background:**

Disordered and hypomineralized woven bone formation by dysfunctional mesenchymal stromal cells (MSCs) characterize delayed fracture healing and endocrine –metabolic bone disorders like fibrous dysplasia and Paget disease of bone. To shed light on molecular players in osteoblast differentiation, woven bone formation, and mineralization by MSCs we looked at the intermediate filament desmin (DES) during the skeletogenic commitment of rat bone marrow MSCs (rBMSCs), where its bone-related action remains elusive.

**Results:**

Monolayer cultures of immunophenotypically- and morphologically - characterized, adult male rBMSCs showed co-localization of desmin (DES) with vimentin, F-actin, and runx2 in all cell morphotypes, each contributing to sparse and dense colonies. Proteomic analysis of these cells revealed a topologically-relevant interactome, focused on cytoskeletal and related enzymes//chaperone/signalling molecules linking DES to runx2 and alkaline phosphatase (ALP). Osteogenic differentiation led to mineralized woven bone nodules confined to dense colonies, significantly smaller and more circular with respect to controls. It significantly increased also colony-forming efficiency and the number of DES-immunoreactive dense colonies, and immunostaining of co-localized DES/runx-2 and DES/ALP. These data confirmed pre-osteoblastic and osteoblastic differentiation, woven bone formation, and mineralization, supporting DES as a player in the molecular pathway leading to the osteogenic fate of rBMSCs.

**Conclusion:**

Immunocytochemical and morphometric studies coupled with proteomic and bioinformatic analysis support the concept that DES may act as an upstream signal for the skeletogenic commitment of rBMSCs. Thus, we suggest that altered metabolism of osteoblasts, woven bone, and mineralization by dysfunctional BMSCs might early be revealed by changes in DES expression//levels. Non-union fractures and endocrine – metabolic bone disorders like fibrous dysplasia and Paget disease of bone might take advantage of this molecular evidence for their early diagnosis and follow-up.

## Introduction

Delayed fracture healing leads to non-unions (NU) in 2 - 5% of traumatic bone lesions, needs at least six months of clinical observation to be diagnosed, and no serum proteins are currently available for an early diagnosis (1). Similar, fibrous dysplasia (FD) and Paget’s disease of bones (Paget) account for 0.05 - 3% of subjects in the general population including children, only minimally relying on blood markers of bone remodeling for diagnosis and assessment of disease activity, becoming useless in cases of low turnover disease (2, 3).

All these disorders have in common an altered bone metabolism characterized by the formation of irregular, weakened, and hypomineralized woven bone (4–6). Though a number of genetic and molecular steps of their pathogenesis are well known, a common cellular feature is the derangement of osteogenesis by mesenchymal stromal cells (MSCs) (7–9). Thus, knowledge of still unrecognized players of altered osteoblast differentiation, woven bone formation, and binding of calcium to the extracellular matrix (ECM) during MSCs skeletogenesis might represent a useful tool for the diagnostics and follow-up of surgical and medical treatment of skeletal conditions like NU, FD, and Paget.

The Type III intermediate filament desmin (DES) has been identified in human bone marrow-derived MSCs (BMSCs) including the congener human dental pulp stem cells, where it is believed a putative marker of multipotency (10–12). Similarly, DES has been identified in MSCs from the perivascular Warthon’s jelly of the human umbilical cord (13–15), responding to osteogenic induction (16, 17). It has also been found in rat BMSCs (rBMSCs) (18–20), mouse mesodermal precursors from embryonic stem cells exhibiting a gene expression profile similar to BMSCs (21), and mouse osteoblasts differentiated from BMSCs (22). Thus, both in humans and rodents, this classical myogenic protein emerges as a part of the molecular machinery of osteogenesis by BM osteoprogenitors. In addition, DES-positive osteogenic cells have been found in the mouse (23, 24) and human skeletal muscle, where they have been suggested as a source of osteoprogenitors involved in ectopic muscle ossification during fibrodysplasia ossificans progressiva (25), although in this progressive heterotopic ossification, endothelial-derived MSCs play a primary role (26), as in traumatic//inflammatory myositis ossificans (27). Consistently, DES expression has been found in vascular derivates of hematopoietic stem cells like human pericytes and endothelial precursor cells (28), the latter favoring increased DES expression when co-cultured with human periodontal ligament fibroblasts in osteogenetic medium (29). Similarly, DES immunostaing has been identified in congener perisinusoidal subendothelial stellate cells of the rat liver (30), human fetal liver (31), and human adult pancreatic islets (32), all exhibiting skeletogenic properties (30, 32). Collectively, DES is readily detectable in different human and rodent BM progenitors retaining osteogenic plasticity, where it seemingly increases during osteogenic commitment.

Finally, a key role for DES in bone formation is suggested by the stromal interaction molecule 1 (STIM1) R304W gain-of-function knock-in mouse model, that recapitulates the main clinical features observed in patients with tubular aggregates myopathy (TAM; OMIM #160565 and #615883) and Stormoken syndrome (STRMK; OMIM #185070). Like the R304W STIM1-mutated mouse, TAM and STRMK patients have overactivation of the Store-operated Ca^2+^ entry (SOCE) system, through dominant STIM1 and Ca^2+^ release-activated Ca^2+^ (CRAC) channel ORAI1 gain-of-function mutations (33). As a result, excess calcium enters the cell leading to intracellular hypercalcemic stress, and the inability to use calcium for mineralization. Consistently, abnormal bone development, growth, and architecture occur in the targeted transgenic mouse (33, 34) and anecdotally in cases of TAM and STRMK (35, 36). Since in non-excitable cells DES blunts SOCE by restraining STIM1 access to ORAI1, it may act as a cytoskeletal controller of intracellular Ca^2+^, and loss of DES control over STIM1 diffusion along the endoplasmic reticulum has recently been hypothesized in the pathogenesis of TAM (37). Therefore, increased DES in osteogenesis might serve to prevent intracellular hypercalcemia while ensuring adequate extracellular Ca^2+^ for mineral deposition. Conversely, the absence of DES increase would suggest hampered BMSCs osteogenesis.

Having this target in mind, we decided to explore the potential relevance of DES during *in vitro* skeletogenic commitment, woven bone formation, and mineralization by rat BMSCs. These rodent osteoprogenitors have intrinsic immunomodulatory properties like their human counterpart (38), exhibit a stable immunophenotype at early culture passages (39), have morphotypes and replication features similar to human MSCs (40), and give rise to a disordered and not remodeled woven bone (41) mimicking the disordered and weakened osteoid observed in NU, FD, and Paget (4–6).

## Materials and methods

### Animals, isolation of rat bone marrow-derived mesenchymal stromal cells, and culture of C2C12 mouse myoblasts

Twelve 225-250 g (7-8 weeks old) Sprague-Dawley male rats (Charles River, Italy) were used as BM donors. All animal studies were conducted in accordance with the guidelines of the Institutional Ethical Committee for Animal Use in Research of the University of Parma, and the Italian Ministry of Health (approval code 18/2016-PR), Italy. Animal numbers were chosen based on a previous power analysis (see section on Statistical Analysis). rBMSCs were isolated and expanded as recently reported (20, 42, 43). Technical details including microscopy evaluation are available in **Supplementary Data**. C2C12 multipotent mesenchymal myoblasts (ECCC, n. 91031101), a subclone of the C2 mouse cell line (44) were cultured in T25 flasks with D-MEM-high glucose (HG) supplemented with 15% FCS. To avoid myoblast differentiation and get a molecular insight into the multipotent mesenchymal phenotype, C2C12 were detached at their first subconfluence and used for all the experiments.

### Flow cytometric immunophenotyping of rBMSCs

rBMSCs at P2 were harvested by trypsinization (see above), centrifuged at 220 xg for 10 minutes, resuspended in a culture medium, and counted using a Burker chamber. A solution of 7 x10^4^ cells in 100 microl of a PBS buffer containing 0.1% sodium azide and 2% fetal bovine serum (FBS) was used for each flow cytometric point, and cells were incubated at 4°C for 15 min with monoclonal antibodies against rat CD45, CD73, CD90 (BD Pharmingen, USA), as we previously reported (20). Technical details of immunolabeling and cytometric procedure and analysis are given in **Supplementary Data**.

### Light microscopic immunofluorescence, immunocytochemistry, and histochemistry of rBMSCs

For light microscopic (LM) single-labeling indirect immunofluorescence (IF), cells were initially immunostained using either a polyclonal rabbit anti -DES (1:50, Abcam, UK) for 1h at RT, or a monoclonal mouse anti-vimentin (1:500, clone VIM 13., SIGMA, USA), or a rat anti-human RUNX2/CBFA1 to the isoform 2 (1:30, R&D Systems, USA) cross-reacting with rat, overnight at 4C°. Tetramethylrhodamine isothiocyanate (TRITC)-conjugated or fluorescein isothiocyanate (FITC)-conjugated secondary antibodies were used.

For LM double-labeling IF, rBMSCs were initially immunostained for DES (see above), followed by monoclonal antibodies to CD45, CD73, and CD90.

For LM single-labeling immunocytochemistry (IC), rBMSCs were initially immunolabeled for DS (see above), and the reaction product was visualized using an ABC kit (Vectastain), with diaminobenzidine (DAB) as a chromogen.

For combined LM, double-labeling IF/IC rBMSCs were initially immunostained for DES (see single-labeling IF protocol), then immunolabeled with a monoclonal antibody to alkaline phosphatase (ALP), and the reaction product developed using an ABC kit (Vectastain), with DAB as a chromogen.

For combined LM double-labeling histochemistry (HC)/IC, rBMSCs were initially stained for calcium deposition with alizarin red, then immunostained for DES (see above).

For LM fluorescent HC, rBMSCs were stained using TRITC-coupled phalloidin (P1951, Sigma).

Technical details of all the techniques summarized above including type of microscopy evaluation and acquisition of images are provided in **Supplementary Data**.

### Scanning and transmission electron microscopy

Three-dimensional (3D) morphology of rBMSCs was analyzed by scanning electron microscopy (SEM), as previously described (20). Similarly, the subcellular organization of rBMSCs morphotypes was studied by transmission electron microscopy (TEM), as previously described (20). Technical details of these procedures are found in **Supplementary Data**.

### Western blot analysis of desmin in rBMSCs

For Western blotting, lysed rBMSCs were used for protein extraction, and denatured proteins were blotted with anti-DES antibody (see above) on SDS-PAGE. β-actin was recognized as a reference standard through a polyclonal antibody. Immunoreaction products were developed using HRP-conjugated secondary antibodies. Immunoblotted bands were analyzed by semiquantitative densitometry. Technical details of these procedures are found in **Supplementary Data**.

### Preparation of rBMSCs for mass spectrometry analysis, and LC-LIT-Orbitrap XL qualitative mass spectrometry

rBMSCs at P4 were seeded at 7 x10^3^ cells/cm^2^, and cultured for 3 days in rat tail collagen-coated, 6 well polystyrene dishes (Euroclone, ET3006) using DMEM-HG with 10% FCS, 1% P/S, 1% non-essential amino acids, 1% glutamine, and 20μI/100mI of 50 mg/ml gentamicin solution. Rat tail collagen was extracted and chloroform-sterilized following a protocol recently developed by our group (43), 1% collagen solution was put in each culture well and excess liquid was drained off, the bottom collagen layer was left to dry overnight under UV light in a laminar flow hood, and additionally sterilized with 2% P/S and 40μI/100mI of 50 mg/ml gentamicin for 24 hs. Once grown, the cells were detached, proteins quantified, reduced, alkylated, and finally digested to yield tryptic fragments. Then samples were prepared for loading, washed, dried in nitrogen, and reconstituted. Finally, tryptic separation of the digested proteins was carried out by HPLC. Then, qualitative protein analysis was obtained by mass spectrometry (MS) using an LTQ linear ion trap-Orbitrap XL instrument (ThermoScientific Corporation, San José, CA, USA). Data were obtained from two separate samples. All raw data were deposited in the Repository of “Centro Misure Giuseppe Casnati”, UNIPR, Parma, Italy to be available upon motivated request. Technical details of these procedures are found in **Supplementary Data**.

### Data analysis and bioinformatics

The raw data from the Orbitrap Fusion were visually analyzed with Xcalibur™ software, whereas MaxQuant computational proteomics platform (version 1.6.2.10) was used for processing the data. Technical details of these procedures are found in **Supplementary Data**.

Analysis of the biological relevance of data from MaxQuant was made with Perseus (version 1.6.1.1). In order to facilitate the calculation of the protein’s expression level, the raw values of each sample were transformed to a Log2 scale. In the case of Non Assigned Number values in one sample, valid values were considered those of the other sample. Gene Ontology analysis for biological processes and molecular function annotations was performed with Perseus and Search Tool for the Retrieval of Interacting Genes/Proteins (STRING) v10.5.

### Construction and topological analysis of the protein-protein interaction network of desmin-mediated osteogenesis

Construction of the protein-protein interaction (PPI) network focused on a putative DES-mediated osteogenetic pathway was primarily based on the list of proteins experimentally detected at MS, and known to be relevant to the cytoskeletal machinery. Of these, we selected the proteins known to be involved in osteogenic differentiation, including the two master genes runx2 and ALP (45–48). Using the database and algorithm of STRING v12, a PPI undirected graph of nodes, edges, and paths was achieved on the assumption of neighbor interactors in curated STRING databases at a very high level of confidence (score 0.9).

To analyze the structure of the ensuing PPI network, the Open-Source Gephi 0.10.1 program for large network analysis (see at http://gephi.org) was used (49). Calculated topological indexes included: *node degree* (k), defined as the number of edges linked to each node; *between centrality of a node* (BC), defined as the proportion of the number of shortest paths passing through it to all the shortest pathways in the network; *eccentricity* (Ecc), defined as the the maxium distance from the node to all other nodes; *closeness centrality* (CC), defined as the inverse of the average length of the shortest paths between the node and all the other nodes; *average degree of the network* (< k >), defined as the mean of all node degrees in the network; average clustering coefficient (Acc), defined as a measure of the local interconnectedness of the graph. Nodes with the top 10% highest degree (k) and BC were considered the “hub” and the “bottleneck” of the PPI network, collectively identifying the “backbone” of the PPI network (49).

### Osteogenic differentiation of rBMSCs

rBMSCs were differentiated using an osteogenic medium in DMEM-HG, with 50 µM ascorbic acid, 10 mM β-glycerol phosphate, 0.1 µM dexamethasone. The differentiation medium was replaced every 2 days. Colony mineralization and formation of bone nodules were assessed at 14 and 21 days using alizarin red staining (Alizarin Red S A5533, Sigma-Aldrich). Technical details of these procedures including the microscopy protocol are found in **Supplementary Data**.

### Definition of colony and colony density, colony size and circularity, and colony-forming efficiency of rBMSCs

To avoid cells randomly grown in close proximity, we defined as colonies only those cell aggregates visible under LM at x10 –40 and having more than 14 cells, corresponding to more than 1.75 cell doublings, that express the average cell replication kinetic at subconfluence of this cell type, as defined in our previous studies (20, 43). On this basis, colonies were defined as sparse (S) and dense (D) dependent on the countability of cell nuclei, which was achievable only in S colonies, as opposed to D colonies where nuclei resulted too strictly packed for a clear distinction by two independent observers. To evaluate the size and circularity of both S and D colonies, an original methodology was applied, based on images of each colony taken on either LM at x10 –40 or SEM at x1250, in jpeg format. Technical details of this method are available in **Supplementary Data** and rely on well-established mathematical principles (50–56).

The efficiency of seeded cells to give rise to DES-IR D colonies (i.e. CFE) was calculated at LM (see below) as the ratio between the number of colonies in control and differentiated cultures per 1 x10^5^ seeded cells at subconfluence, i.e. immediately before starting the experimental procedure (57)

### Quantitative and semi-quantitative light microscopic analysis of rBMSCs

The number of S and D colonies in control and differentiated cultures was calculated with LM at x100 following the division of an index culture (selected from 3 different samples), in 4 optical quadrants of identical extention. Results were expressed as mean number/optical quadrant of S and D colonies. On a similar index culture, evaluation of CFE in control and differentiated D DES-IR colonies, and of control and DES-IR colony size and circularity were performed using either LM at x10 - 40 or SEM at x1250, on 22, 25, 34 and 25 different optical fields, respectively (for calculation of CFE, colony circularity, and colony size see above). The number of DES-IR D colonies, and single cells IR for DES but outside the colonies, was obtained by an unbiased, grid-based counting method with LM at x100 on 20 different optical fields in control and differentiated cultures, respectively and expressed as percentage of D DES-IR colonies and cells. An evaluation of the intensity of IR staining for DES, runx2, and ALP in D colonies of control and differentiated cultures was performed with LM at x100 on 3 randomly chosen colonies of one index preparation, by 3 independent observers. Results were expressed as the average of the three observations, using an ordinal semiquantitative visual scoring method (58) as follows: - = staining in less than 25% of total cells; + = staining in 25 - 50% of total cells; ++ = staining in 51 - 75% of total cells; +++ = staining in > 75% of total cells.

### Statistical analysis

The group size of animals for the cell culture studies was calculated using the G*Power v.3.1.7 software (Franz Faul, Universität Kiel, Germany). By considering an obtained effect size (d=1.78) with a power of 80% and a value of p < 0.05, we predicted an effect size f = 0,50 leading to a minimum of n = 6 animals/group (two-way analysis of variance or ANOVA for repeated measures). In addition, differences in: a) number of S and D colonies in control and differentiated cultures; and b) size and circularity of S and D colonies in control and differentiated cultures, all were evaluated using a one-way ANOVA, and a Student-Newman-Keuls test on means. Proportion of DES-IR, D colonies and single cells outside the colonies in control versus differentiated cultures was computed using a chi squared test. All the differences were considered significant if p < 0.05.

## Results

### Immunophenotyping, morphological, cytochemical, and molecular characterization of rBMSCs

**Figure 1:**
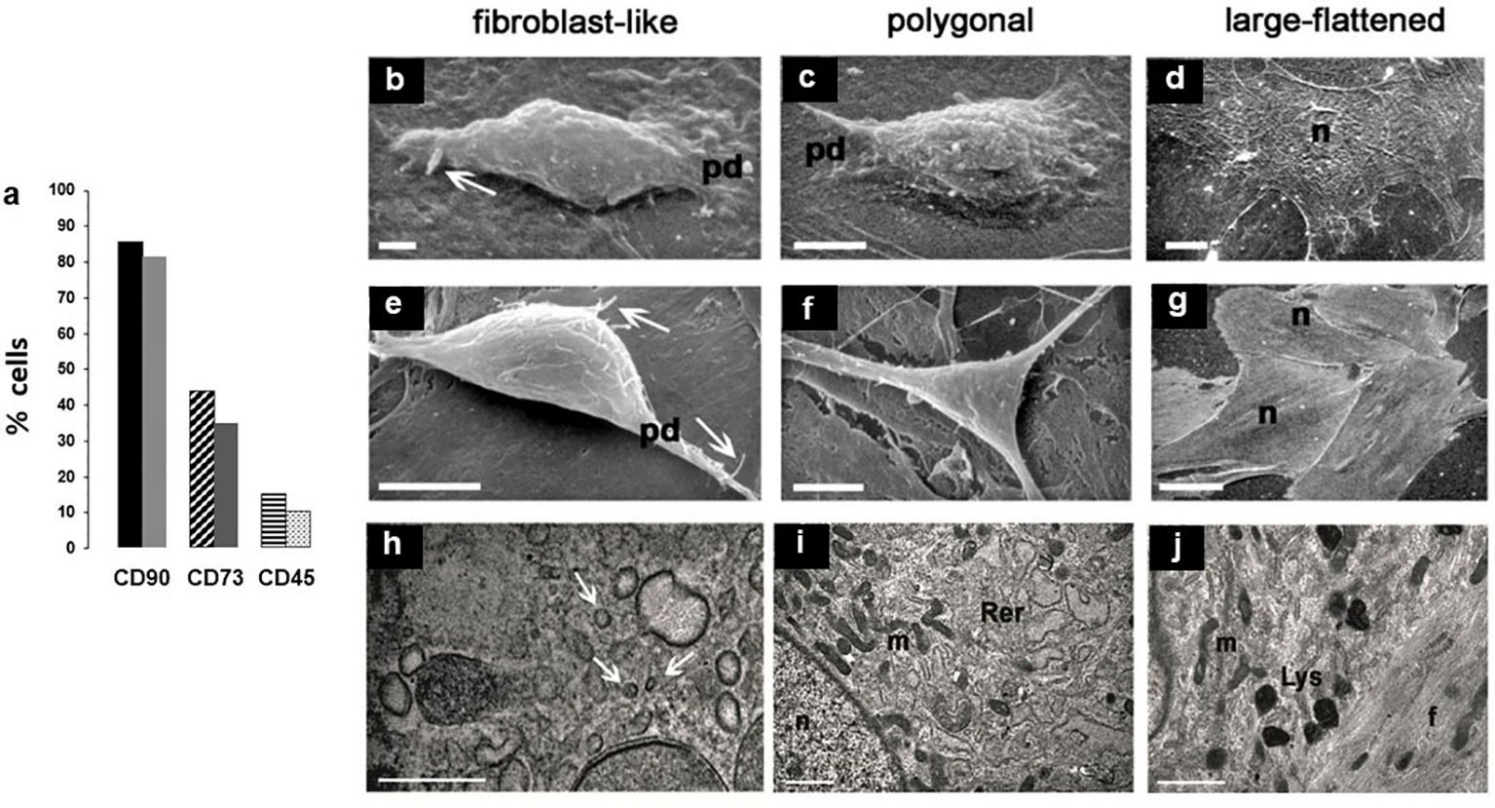
**(A)** Histograms of two separate experiments on rBMSCs immunophenotyping at P2, each histogram showing percentages of IR-cells: IR-CD90 and IR-CD73 cells prevailed on a negligible presence of IR-CD45 cells, confirming adequacy of the isolation procedure for cells of the mesenchymal lineage; **(B-L)** morphotypes of adult male rBMSCs, after 7 - 21 days in monolayer culture. At SEM, three constant morphologies (fibroblast-like, polygonal, large-flattened) were observed, irrespective of cells grown on either **(B-D)** polystyrene plastic or **(E-G)** glass. In fibroblast-like and polygonal phenotypes, well-developed cell surface specializations like pseudopodia (pd) and filopodia (white arrows) suggest contractile and motility properties, as in the presence of functional cytoskeletal proteins. In contrast, in large-flattened phenotypes pavement-like selfassembly demonstrates an increase in the cell area, expected for osteoprogenitors grown on rigid substrates. TEM analysis of rBMSCs grown on glass revealed an ultrastructural organization consistent with that of progenitor cells of the mesenchymal lineage. In particular, **(H)** fibroblast-like cells displayed numerous intracytoplasmic vesicles (white arrows), whereas **(I)** polygonal cells contained abundant and dilated rough endoplasmic reticulum (Rer) and elongated mitochondria (m), similarly present also in **(J)** large-flattened cells harboring numerous lysosomes (Lys) and dense filaments (f), as expected for accumulation of cytoskeletal proteins. Each image represents the result of a sample selected from an average of three different experiments. n = nucleus; bar **(H)** = 0.2 microns; **(B, I, J)** = 1 micron; **(C, E, F)** = 5 microns; **(D)** = 10 microns; **(G)** = 50 micron.

Consistent with our previous studies (20, 43), flow cytometric immunophenotyping of two separate rBMSCs samples yelded 82 - 86% and 35 - 44% of cells immunoreactive (IR) for CD90 and CD73, respectively. In contrast, only 10 - 15% of cells resulted IR for CD45 (**Figure 1A**). This evidence confirmed an immunophenotypic profile compatibile with adult rat stem cells of the MSCs lineage (data collection courtesy of Drs. Alessandra Zamparelli, Grant FIRB RBAP10MLK7_004 post-doctoral Fellowship 2010 –2015 and Luca Cattini, Laboratories of Immunorheumatology and Tissue Regeneration, RAMSES, IOR, Bologna, Italy).

Then, to test the reliability of isolation and expansion of rBMSCs in monolayer culture, we studied their morphotypes. As shown in previous studies (20, 43), scanning electron microscopy (SEM) revealed three constant cell morphologies including fibroblast-like (**Figures 1B, E**), polygonal (**Figures 1C, F**), and large-flattened (**Figures 1D, G**) cells. These morphologies remained substantially unchanged when cells were cultured on either standard polystyrene flasks (**Figures 1B–D**) or glass slides (**Figures 1E–G**), retaining typical surface specializations like pseudopodia and filopodia, and pavement-like self-assembly.

Similarly, as previously reported (20) transmission electron microscopy showed that fibroblast-like cells contained numerous clear vesicles (**Figure 1H**), whereas polygonal cells were characterized by abundant and dilated rough endoplasmic reticulum and elongated mitochondria (**Figure 1I**). In contrast, large-flattened cells exhibited numerous thick and randomly crossing bundles of fibers throughout their cytoplasm, often entrapping enlarged mitochondria, and diffuse lysosomes (**Figure 1J**). We concluded that all cell morphotypes belong to adult progenitors of the same MSCs lineage.

**Figure 2:**
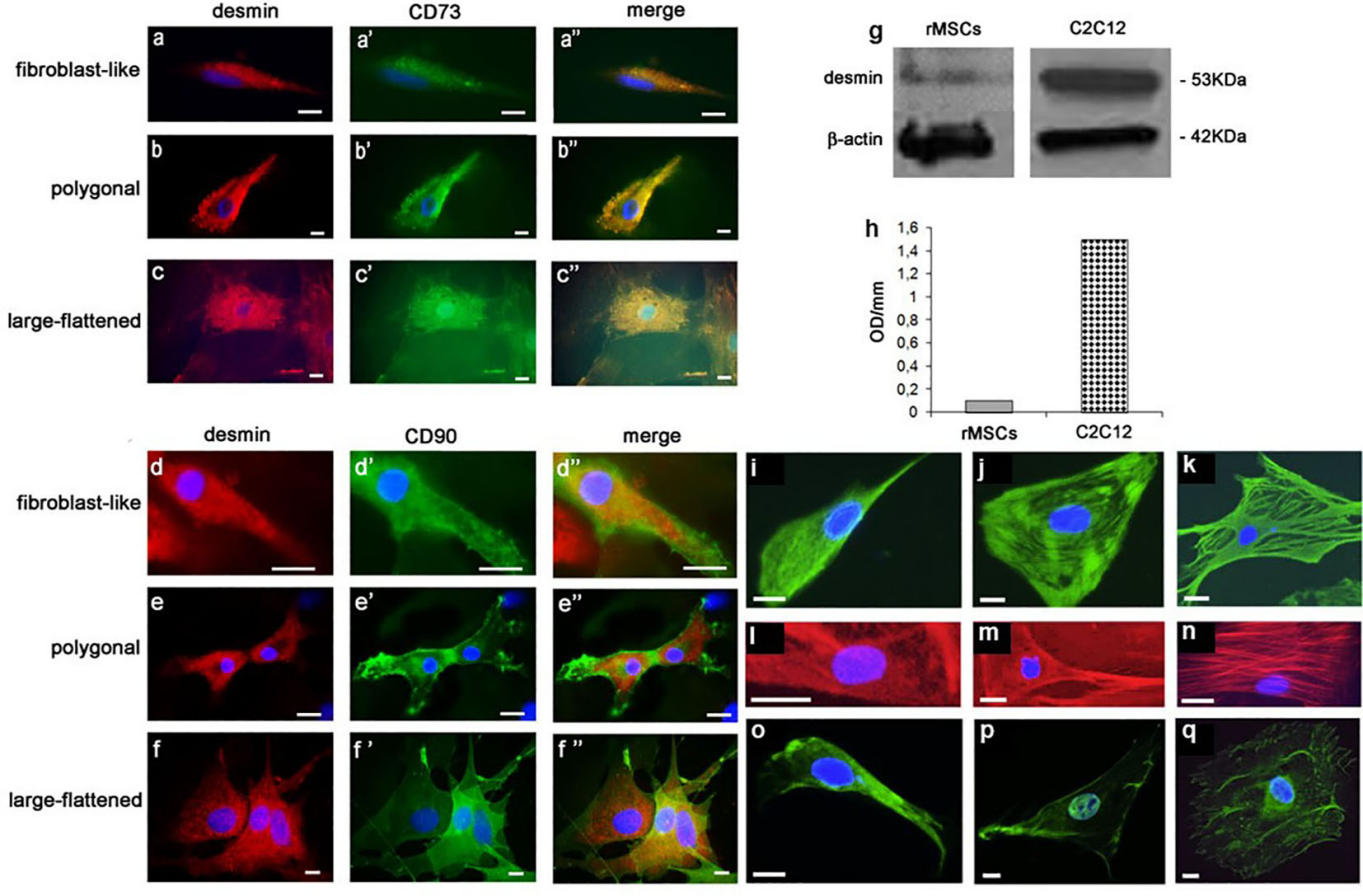
LM IF of DES, CD73, CD90, vimentin, F-actin, and runx2 in rBMSCs at P2 grown on glass for 7 –14 days, and their DES Western blotting (WB) with respect to control C2C12 myoblasts. Using double-labeling, IR-DES was found to co-localize with IR-CD73 and IR-CD90 in all cell morphotypes including **(A - A’’, D-D’’)** fibroblast-like, (**B-B’’, E-E’’)** polygonal, and **(C-C’’, F-F’’)** large-flattened cells. Note its different organization in relation to the cell morphotype, where fibroblast-like cells diffuses along the cell longitudinal axis, polygonal cells predominates in the perinuclear position, and large-flattened cells spread throughout the cytoplasm. No IR-CD45 cell resulted positive for DES (data not shown). The authenticity of the DES molecule was confirmed by WB: **(G)** montage of the protein bands relevant to both cell types, including one of 53 kDa corresponding to DES, and another of 42 KD related to β-actin as a control; **(H)** content of DES, as determined by semiquantitative densitometric analysis, revealing authentic DES in both cell types. Each blot represents a single experiment. OD = optical density. In addition, using single-labeling all cell morphotypes showed strong immunostaining also for (green color, **I – K**) vimentin, (red color, **L – N**) F-actin, and (green color, **O – Q**) runx 2, each molecule having a peculiar intracellular topography described in detail in the Results, to some extent reconciling with that of DES. (Courtesy of Drs. Alessandra Zamparelli, Grant FIRB RBAP10MLK7_004 post-doctoral Fellowship 2010- 2015 and Elena Bassi, Feliciani-Ferretti Fund 2013 - 2014 and postdoctoral Fellowships 2014 - 2016, UNIPR, Parma, Italy). Each image represents the result of a sample selected from an average of three different experiments. Bars [(**A – F"**) and (**L – Q**)] = 10 microns; **I – K** = 50 microns.

In a second step, using light microscopic (LM) immunocytochemistry (IC) we studied the cellular topography of desmin (DES) in immunophenotypically-identified cell types. Immunoreactive (IR) - DES (**Figures 2A–F**) was found to co-localize (**Figures **2A’’–F’’) with IR-CD73 (**Figures 2**A’–C’) and IR-CD90 (**Figures **2D’–F’) in all three rBMSCs morphotypes. In particular, in fibroblast-like cells DES was organized along their longitudinal axis (**Figures 2A**, 2A’’, **2D**, 2D’’), in polygonal cells it predominantly gathered in perinuclear position (**Figures 2B**, 2B’’, **2E**, 2E’’), and in large-flattened cells, DES was spread throughout the entire cytoplasm (**Figures 2C**, 2C’’, **2F**, 2F’’). In contrast, no IR-CD45 cells were found to contain IR-DES (data not shown). The presence of authentic DES in all cell morphotypes was confirmed by western blot analysis, using as a positive control the C2C12 myoblast cell line. A band of 53 kDa corresponding to DES was revealed in both cell types (**Figure 2G**), whose densitometric amount resulted about 10 times lower in rBMSCs with respect to myoblasts (**Figure 2H**). Collectively, these data supported the appropriateness of the procedures used to isolate and expand adult male rBMSCs, and the availability of DES in all undifferentiated rBMSCs, with patterns of distribution depending on the cell morphotype.

In a third step, we examined the cell distribution of key cytoskeletal and differentiation markers involved in osteogenesis including vimentin, F-actin, and runx2. In fibroblast-like cells, a network of very thin IR-vimentin filaments filled the entire cytoplasm (**Figure 2I**) as opposed to polygonal cells, where parallel and concentric filaments organized in the cytoplasm towards the nucleus, and to some extent compacted underneath the plasma membrane (**Figure 2J**). Differently, the cytoplasm of large-flattened cells was filled with coarse and dense protein bundles, assuming a crossed and random distribution along its major axis (**Figure 2K**).

Equally, in fibroblast-like cells fine phalloidin-stained F-actin filaments occupied the entire cytoplasm (**Figure 2L**). In contrast, in polygonal cells actin accumulated in the perinuclear position and underneath the cell membrane, leaving the rest of the cytoplasm relatively free of immunoreactivity (**Figure 2M**). Differently, in large-flattened cells, the cytoplasm was filled with well-organized actin fibers coursing partly parallel to the main cell axis, and crossing each other (**Figure 2N**).

Finally, a search for the master regulator of preosteoblast differentiation runx2 showed that it diffusely localized in the cytoplasm of fibroblast-like cells (**Figure 2O**), as opposed to its prominent intranuclear segregation in polygonal cells (**Figure 2P**), and to a perinuclear and scanty fiber-like presence in the cytoplasm of large-flattened cells (**Figure 2Q**). Collectively, these results showed a topographic relation between mechanotransduction and the osteogenic machinery, reciprocally modifying dependently on the rBMSC morphotype.

### Cytoskeletal proteomic profile, and protein-protein interaction network involving desmin in the skeletogenic commitment of rBMSCs

**Figure 3:**
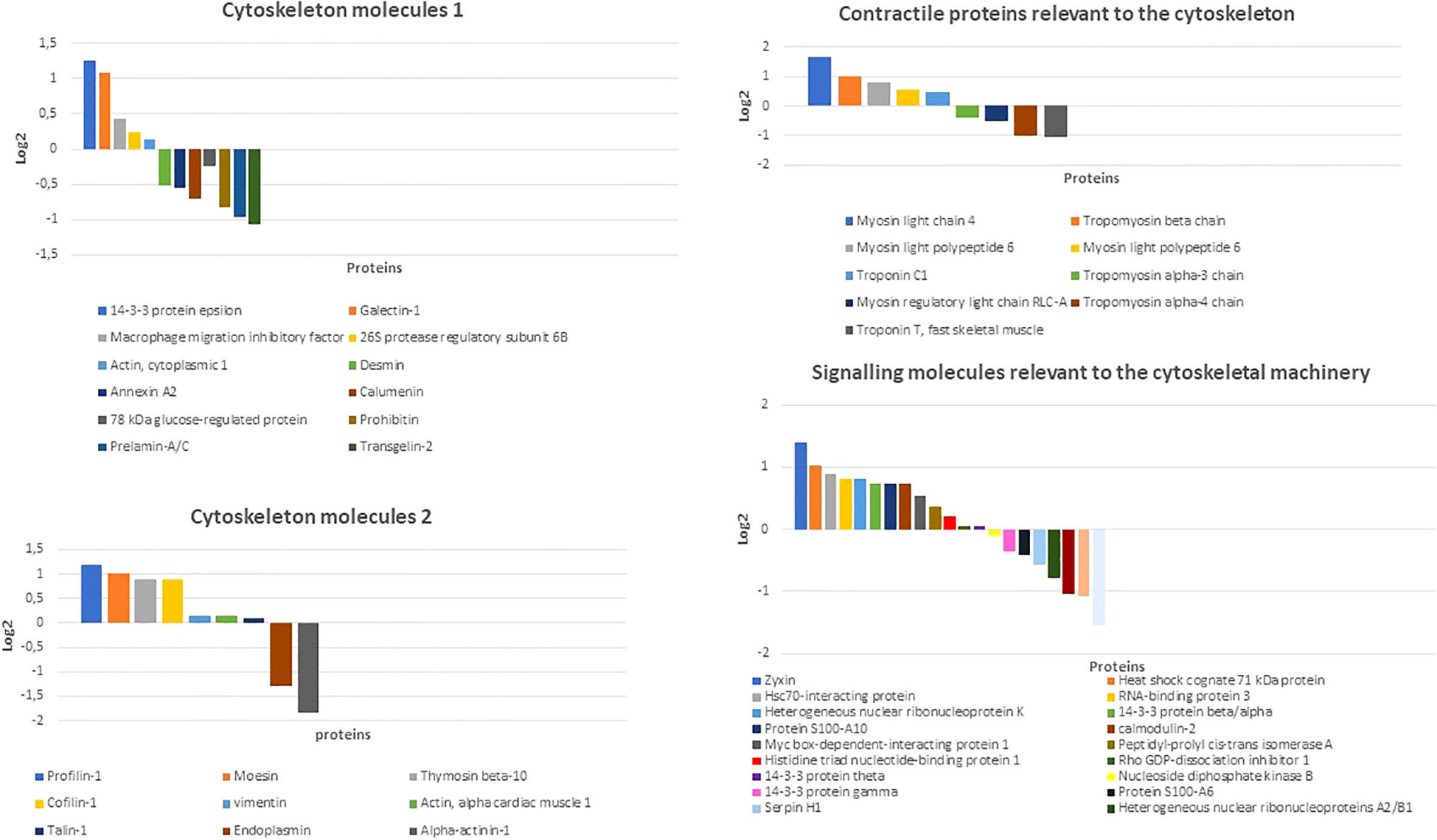
Distribution of main cytoskeletal, contractile, and related signaling molecules of rBMSCs as detected by qualitative mass spectrometry (MS). Note the presence of an array of proteins known to interact (either directly or indirectly) with DES and contribute to the osteogenic process and immunomodulatory properties including vimentin, actins, calumenin, transgelin-2, annexin-2, prohibitin, galectin-1, profilin-1, cofilin-1, talin-1, moesin, thymosin beta10, prelamin - A/C, myosins, troponins, tropomyosins, and zyxin. Amongst signaling molecules related to the cytoskeletal machinery Rho GDP-dissociation inhibitor 1, calmodulin-2, myc box-dependent-interacting protein 1, and proteins S-100 emerged as possible partners for an osteogenetic role of DES. Other cytoskeletal, chaperonic, and enzymatic interactors included tubulin, vinculin, heat shock protein beta-1 or Hsp27, Hsp60, Hsp70, Hsp90, and cytoplasmic aspartate aminotransferase (see **Supplementary Figures 1**). Data were collected from two independent rBMSCs samples, and here shown the results from the sample with the best Log 2 values. (Courtesy of Drs. Elia Consolini, PhD Program in Molecular Medicine 2016-2019 and Andrea Faccini, “Centro Misure Giuseppe Casnati”, UNIPR, Parma, Italy).

To get hints on the cytoskeletal machinery of rBMSCs, we used proteomic analysis. Qualitative mass spectrometry (MS) of two separate samples of rBMSCs, and related functional clustering by Gene Ontology focused on molecules of the cytoskeleton, cellular contractility, chaperones, and connected signaling processes revealed a total of 85 proteins including DES, among which those known to be involved in osteogenesis were vimentin, actins, profilin-1, cofilin-1, talin-1, moesin, tubulin, vinculin, thymosin beta10, prelamin - A/C, myosins, troponins, tropomyosins, heat shock protein beta-1 or Hsp27, Hsp10, Hsp60, Hsp70, Hsp90, and zyxin. Additional molecules linking the cytoskeletal machinery to both the skeletogenic and immunomodulatory activities included calumenin, transgelin-2, annexin-2, prohibitin, and galectin-1. Similarly, signaling molecules involved in different cytoskeletal actions comprised the Rho GDP-dissociation inhibitor 1, cytoplasmic aspartate aminotransferase, calmodulin-2, myc box-dependent-interacting protein 1, and proteins S-100 (**Figure 3**, and **Supplementary Figure 1**).

**Figure 4:**
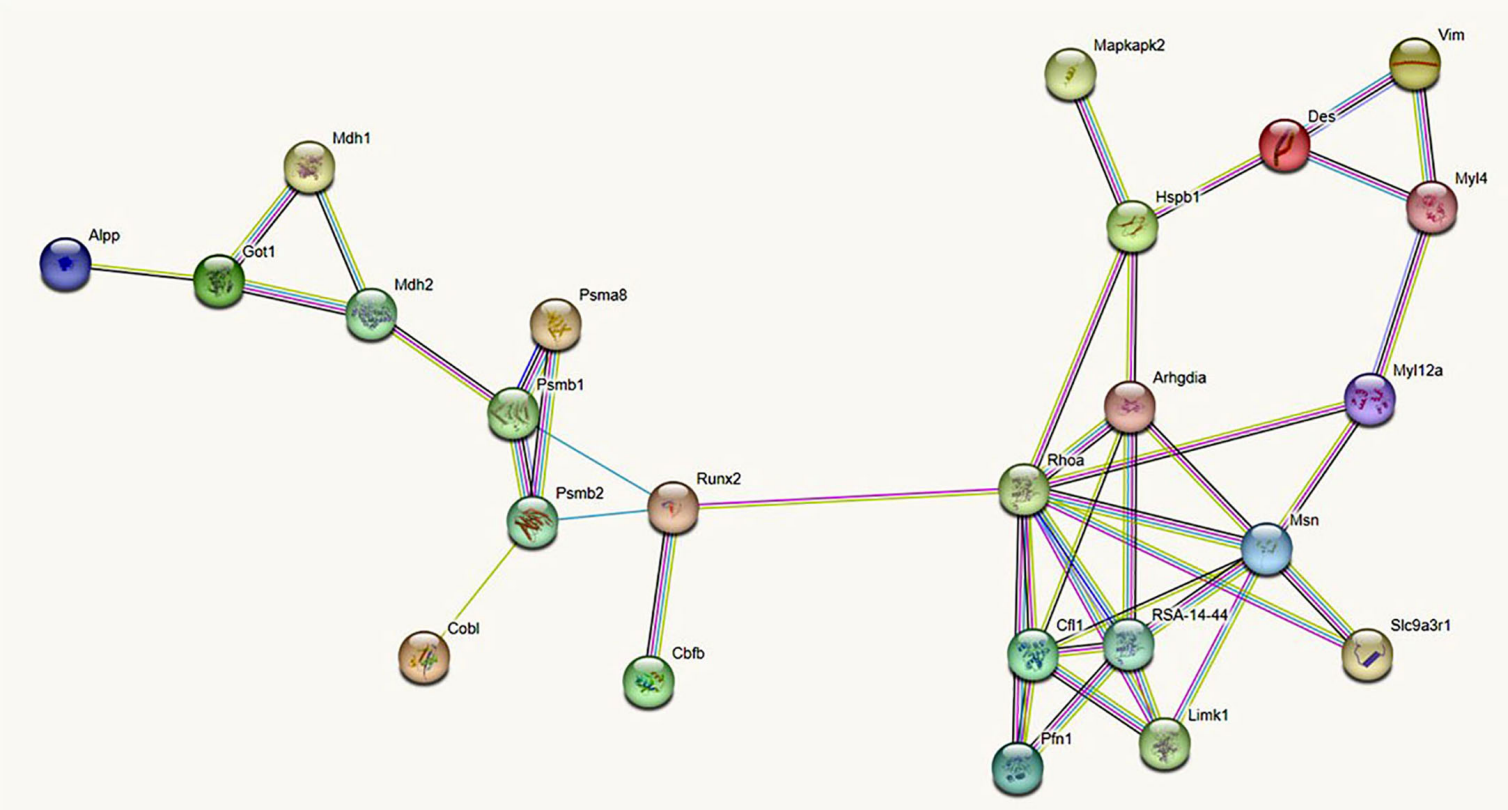
Undirected protein -protein interaction (PPI) STRING network linking DES to 13 rBMSCs proteins (each represented by a circle or node) selected from the MS proteome, after numerous virtual simulations at a progressively minor number of interactors, to ensure an uninterrupted chain of connections. To all cytoskeletal proteins with actions relevant to the rBMSCs skeletogenic commitment, we added the two master regulators runx2 and alkaline phosphatase (ALP). In addition, the STRING algorithm completed the network with 10 functional partners predicted at a very high level of confidence (score 0.9). Note that DES stands as a point of initiation (i.e. a starting node) in the flow of interactions (i.e. the so-called edges of the graph) leading to runx2 and ALP. This supports a role for DES as an early marker of the rBMSCs osteogenic fate. Different colors of the interaction lines (i.e. of the graph edges) signify their sources; light blue: curated databases; pink: experimental determination for known interactors; green: gene neighborhood; red: gene fusion; blue: gene co-occurrence for predicted partners; yellow: text mining; black: co-expression; violet: protein homology for other associations. All proteins refer to the *Rattus Norvegicus* database. Alpp, Alkaline phosphatase; Arhgdia, Rho GDP-dissociation inhibitor 1; Cbfb, core-binding factor, beta subunit; Cfl1, Cofilin-; Cobl, Cordon-bleu wh2 repeat protein; Des: Desmin; Got1, Aspartate aminotransferase, cytoplasmic; Hspb1, Heat shock protein family b (small) member 1; Limk1, Lim-domain kinase 1; Mapkapk2, Mitogen-activated protein kinase-activated protein kinase 2; Mdh1, Malate dehydrogenase, cytoplasmic; Mdh2, Malate dehydrogenase, mitochondrial; Myl4, Myosin, light chain 4; Myl12a, Myosin regulatory light chain RLC-A; Msn, Ezrin-radixin-moesin (ERM) family protein; Pfn1, Profilin-1; Psma8, 20S proteasome subunit alpha 4; Psmb1, 20S proteasome subunit 6; Psmb2, proteasome subunit beta type-2; Rhoa, Ras homologue gene family member a; RSA - 14 - 44, RSA - 14 - 44 protein, GTPase Rho family; Runx2, Runt-related transcription factor 2; Slc9a3r1, Na(+)/H(+) exchange regulatory cofactor NHE-RF1; Vim, vimentin.

**Figure 5:**
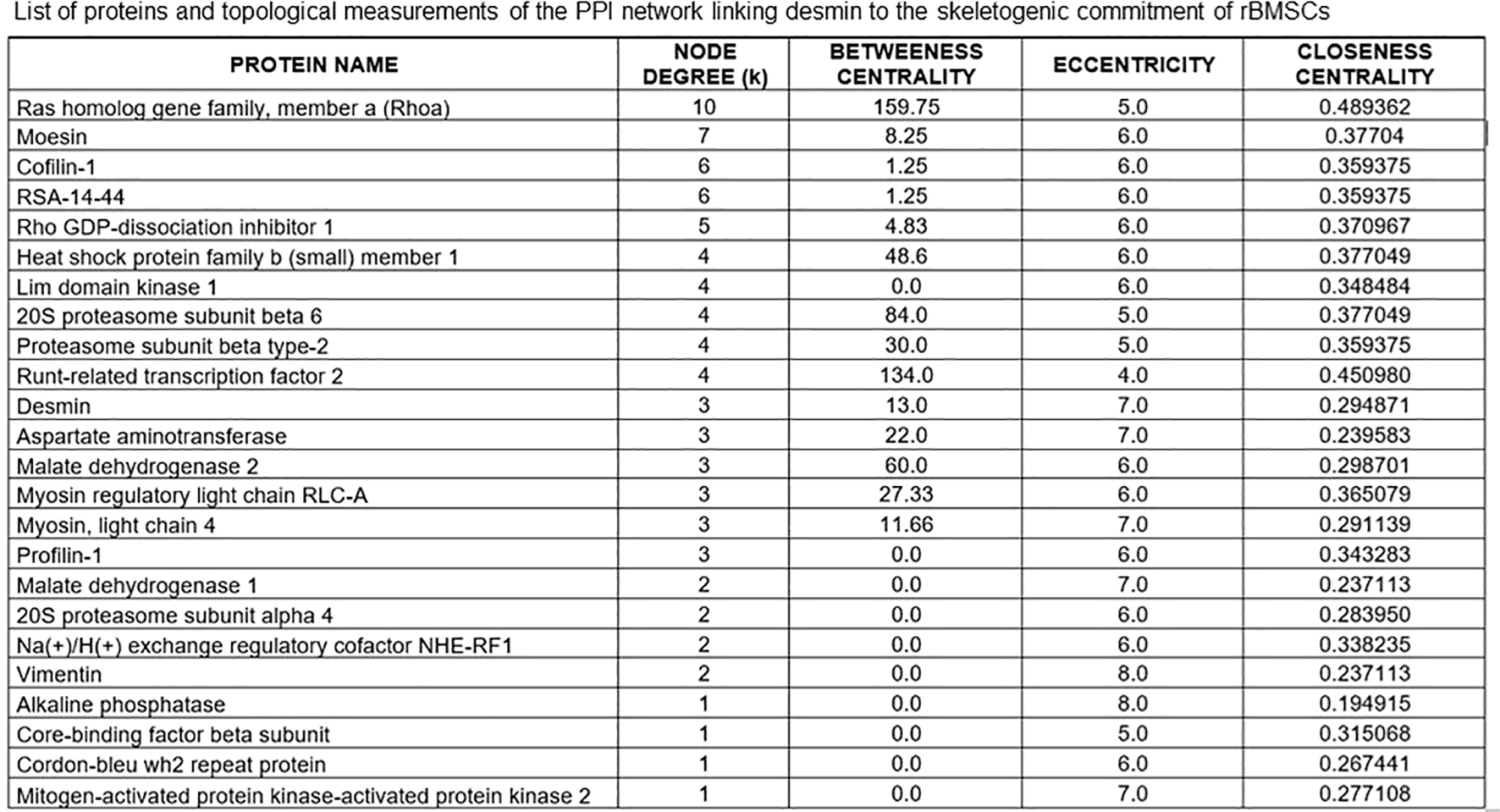
List of proteins of the STRING interactome involving DES in the skeletogenic commitment of rBMSCs, and related topological measurements. Note that the highest node degrees, i.e. the primary sites for convergence of different PPIs, pertain to the Ras homolog gene family member a (Rhoa) and the cytoskeletal protein moesin, which represent the “hubs” of the interactome. However, DES has one of the top 10% highest values for degree k, confirming its role as a key molecular station in the osteogenetic pathway of rBMSCs. Note that Rhoa holds with runx2 also the highest between centrality (BC) and closeness centrality (CC) values while having the lowest eccentricity (Ecc) values. Thus, both proteins may serve as regulatory switches or “bottlenecks” for information flowing throughout the network, whose they also provide the functional “backbone” with moesin. In contrast, DES has lower BC and CC and higher Ecc values, remaining located upstream of both arms of the Rhoa - moesin portion of the backbone pathway and thus, functioning as an early/initial signal for the global interactome. In this interactome, the contractile myosins and proteasome complex emerged as additional regulatory steps, displaying the top 10% highest values for BC.

Based on these MS data, and the inclusion of runx2 and alkaline phosphatase (ALP) as markers of preosteoblastic and osteoblastic differentiation, we constructed an undirected (i.e. not constrained by specific directions) protein-protein interaction (PPI) network or interactome graph, using a heuristic of sequential virtual STRING simulations at progressively more limited number of interactors, to ensure an uninterrupted chain of connections. Starting from a total of 29 relevant molecules, we selected 14 key seed proteins to give rise to a 24-node graph with 42 edges (**Figure 4**), including 10 predicted functional partners at a very high level of confidence (score 0.9). Topological analysis (**Figure 5**) revealed that amongst the top 10% highest values for degree k, Ras homolog gene family member a (Rhoa) and the cytoskeletal protein moesin were preeminent, both representing “hubs” of the PPI network, i.e. primary sites for neighbors interactions. Similar preeminence for between centrality (BC) values converged again to Rhoa, and to a minor extent to runx2, both proteins being the “bottlenecks” of the interactome, i.e. the regulatory switch for information flowing throughout the network. Collectively, these three proteins constituted the “backbone” of the network, though DES had amongst the top 10% highest k values but being located upstream of both arms of the Rhoa –moesin portion of the backbone pathway, and with a low BC value. Remarkably, Rhoa and runx2 also displayed the lowest eccentricity (Ecc) and highest closeness centrality (CC) values, confirming their major functional role in the DES-related osteogenic network. In contrast, DES resulted in higher Ecc and lower CC, revealing its side role in the PPI network, likely as an early/initial activator of the global interactome. The latter resulted in a quite linear informational pathway, with a low number of interactors at each step (average node degree = 3.5) but a robust local interconnectedness consistent with linkages proper to proteins of reciprocal biological relevance (average clustering coefficient = 0.653).

### Colony types, mineralization, colony geometry, desmin content, and co-localization of desmin with runx2 and alkaline phosphatase during osteogenic differentiation of rBMSCs

**Figure 6:**
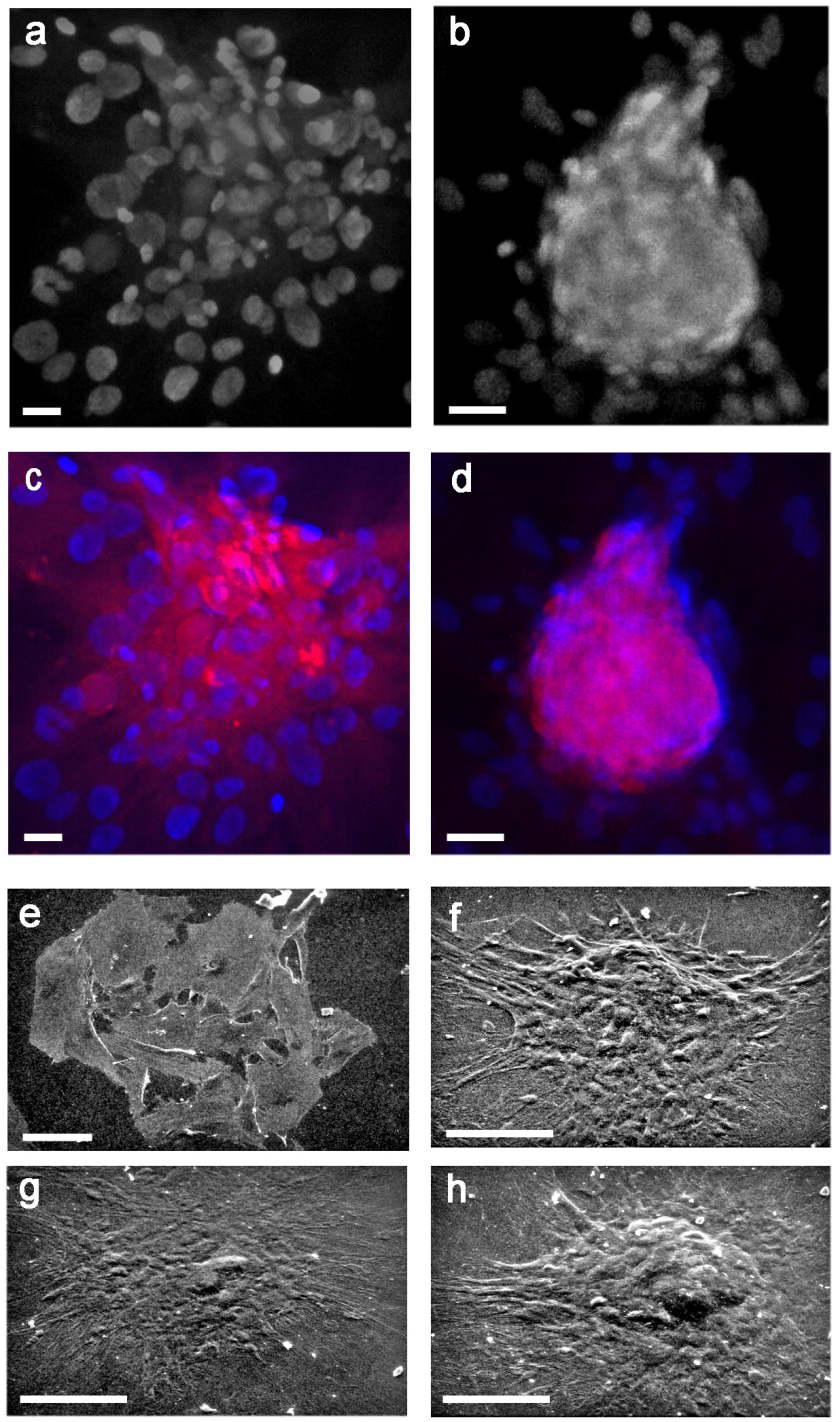
Morphology of control, sparse (S), and dense (D) colonies from rBMSCs at P2, and their DES-IF; **(A)** LM of an S colony, grown on glass for 7 days; **(B)** LM of a D colony, grown on glass for 7 days; **(C, D)** IR-DES in both S and D colonies grown on glass for 7 days; **(E)** SEM of an S colony, grown on glass for 7 days; **(F)** SEM of a D colony, grown on glass for 7 days; **(G)** SEM of an S colony, grown on polystyrene plastic for 21 days; **(H)** SEM of a D colony grown on polystyrene plastic for 21 days. Note the absence of relevant differences in colony morphologies in relation to the growing substrate. Each image represents the result of a sample selected from an average of three different experiments; Bars: **(A-D)** = 20 microns **(F-H)** = 50 microns; **(E)** = 100 microns.

rBMSCs at P2 spontaneously gave rise to 2 different types of colonies, sparse (S) and dense (D) (**Figures 6A, B**) both resulting in IR for DES (**Figures 6C, D**), as expected by the contribution of all cell morphotypes (see above). Remarkably, SEM analysis revealed that both S and D colonies kept unaltered their 3D organization when cells were grown on either glass (**Figures 6E, F**) or polystyrene dishes (**Figures 6G, H**), confirming a similar capacity of these growth substrates to induce selfassembly of cell colonies, as already shown for the morphology of single cells.

**Figure 7:**
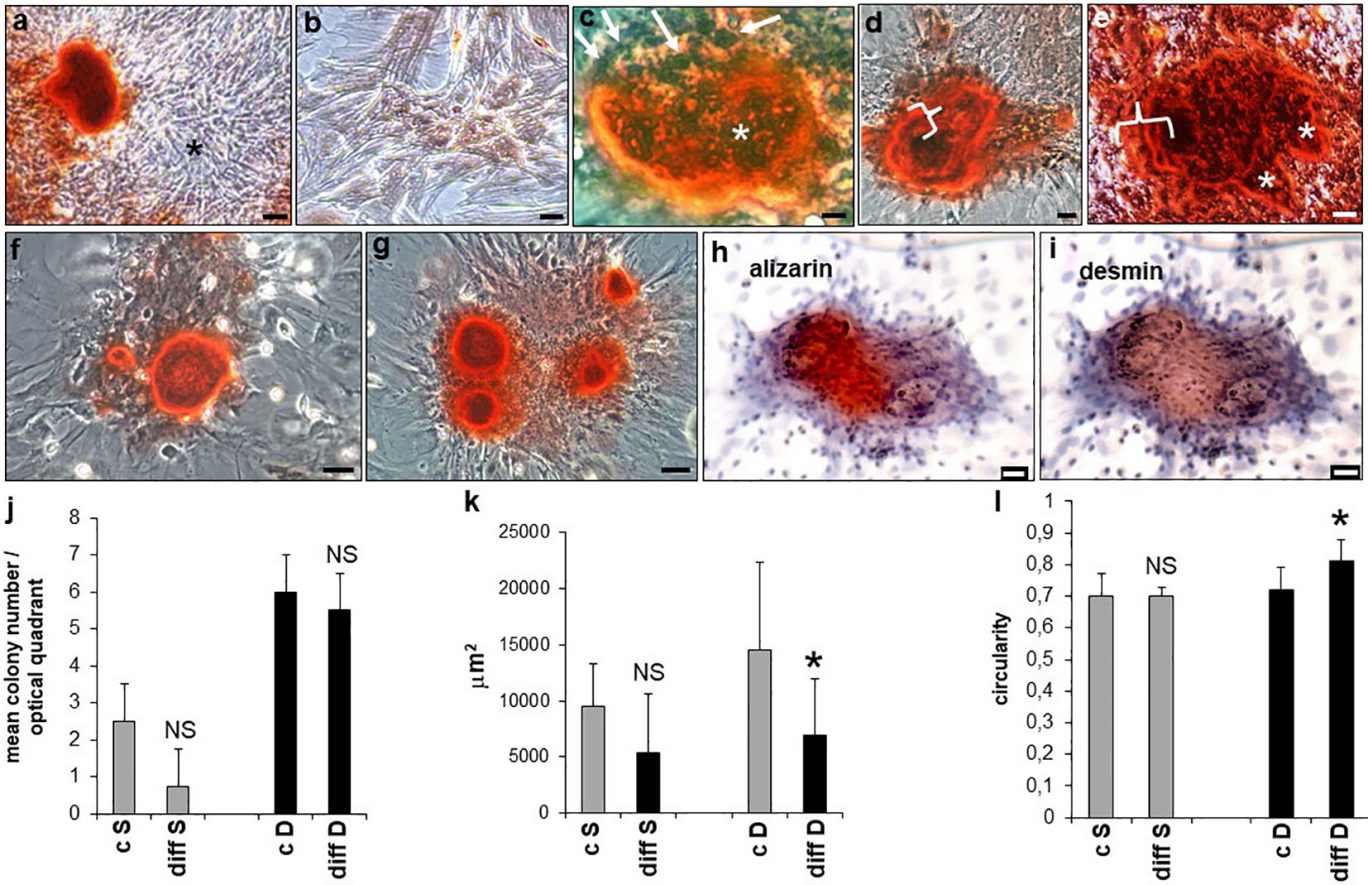
Osteogenic differentiation of rBMSCs at P2, grown on polystyrene plastic: **(A)** at 21 days, calcium nodules (alizarin red staining) were formed only inside D colonies. Note an adjacent unstained S colony (asterisk) devoid of any mineralization; **(B)** negative control; **(C)** at 14 days, calcium appeared deposited at the periphery of the nodule, and surrounded by polygonal dark cells (white arrows) surmounting a layer of amorphous and lightly stained substance, eventually becoming calcified. Polygonal cells (black profiles, asterisk) were embedded in the calcified core (red material) of the nodule; **(D)** once the first line of calcium deposition was completed, a rim of new mineralized tissue was concentrically deposited around (white curly bracket), to give rise to a calcified multilayered structure; **(E)** at 21 days, the bone nodules enlarged by apposition of subnodules (white asterisks) entrapping cells in osteocyte-like lacunae, and showed different and overlapping fronts of calcification (white curly bracket); **(F)** at 14 days, calcium deposits were of limited extent and number (1-2 nodules/colony); **(G)** at 21 days, size and number (4 or more) of calcium deposits increased up to cover the entire colony; **(H-I)** double labeling of a calcified D colony, at 21 days. Note that the area of calcium deposition (alizarin red staining, h) coincides with that of IR-DES DAB brown color, i); **(J)** mean number of S and D colonies in control and differentiated cultures, at 21 days. Osteogenesis did not significantly change the number of both types of colonies, though D colonies were more represented than S colonies; **(K)** mean size of S and D colonies in control and differentiated cultures, at 21 days. Note that only differentiated D colonies were significantly smaller than control colonies; **(L)** mean circularity of S and D colonies in control and differentiated cultures, at 21 days. Note that only differentiated D colonies were significantly more circular than control colonies. Each image represents the result of a sample selected from an average of three different experiments. Each graph represents the result of the same index experiment. (Courtesy of Drs. Alessandra Zamparelli, Grant FIRB RBAP10MLK7_004 post-doctoral Fellowship 2010- 2015 and Elena Bassi, Feliciani-Ferretti Fund 2013 –2014 and post-doctoral Fellowships 2014 –2016, UNIPR, Parma, Italy). Bars **(A)** = 100 microns **(B-I)** = 25 microns; NS, not significant; *p < 0.05.

Following osteogenic differentiation, rBMSCs monolayers were studied at 14 and 21 days revealing the formation of S and D colonies, in both control and differentiated cultures. However, alizarin red distribution showed calcium deposition only in differentiated D but not S colonies (**Figure 7A**). Controls were negative (**Figure 7B**). Mineralization proceeded through the formation of bone nodules of planar circular geometry corresponding to a similar geometry of the D colonies, also showing increased density in their cells. Specifically, at 14 days a ring of calcium was developed inside the majority of D colonies, circumscribed at its surface by a layer of polygonal cells surmounting a ridge of yet uncalcified material. Part of these cells was also recognizably engulfed in the calcified core of the nodule (**Figure 7C**). Once the first line of mineralization was completed, concentric layers of polygonal cells contributed to a calcified multilayered structure (**Figure 7D**). At 21 days, the bone nodules enlarged by the apposition of subnodules entrapping cells in osteocyte-like lacunae, and showed different and overlapping fronts of calcification grossly mimicking the lines of mature osteonic structure (**Figure 7E**). Size and number of calcium deposits progressively increased inside each D colony, from 1 - 2 nodules at 14 days (**Figure 7F**) to 4 and even more nodules at 21 days (**Figure 7G**). Remarkably, double labeling of calcified D colonies revealed that the area of calcium deposition (**Figure 7H**) overlapped that of IR-DES (**Figure 7I**), supporting the concept that DES expression by differentiating rBMSCs was strictly related to their skeletogenic commitment and mineralization of the forming woven bone.

Having observed striking morphological changes in the organization of differentiating D colonies, we proceeded to evaluate the number, size, and circularity of both S and D colonies. Technical details of this procedure are provided in **Supplementary Data** and **Supplementary Figure 2**. Accordingly, at 21 days of differentiation, no statistically significant difference was observed in the number of either S or D colonies between controls and differentiated cells (**Figure 7J**), although D colonies resulted both in control and differentiated cultures in a much higher number with respect to S colonies. In contrast, size and circularity resulted selectively decreased (**Figure 7K**) and increased (**Figure 7L**) in the D colonies of differentiated with respect to control cultures, whereas no statistically significant changes occurred in S colonies. These results indicated that adult male rBMSCs in the control monolayer preferentially gave rise to D colonies, whose number remained stable during their skeletogenic commitment, leading to colony geometrical changes only when mineralization of the osteoid occurred.

**Figure 8:**
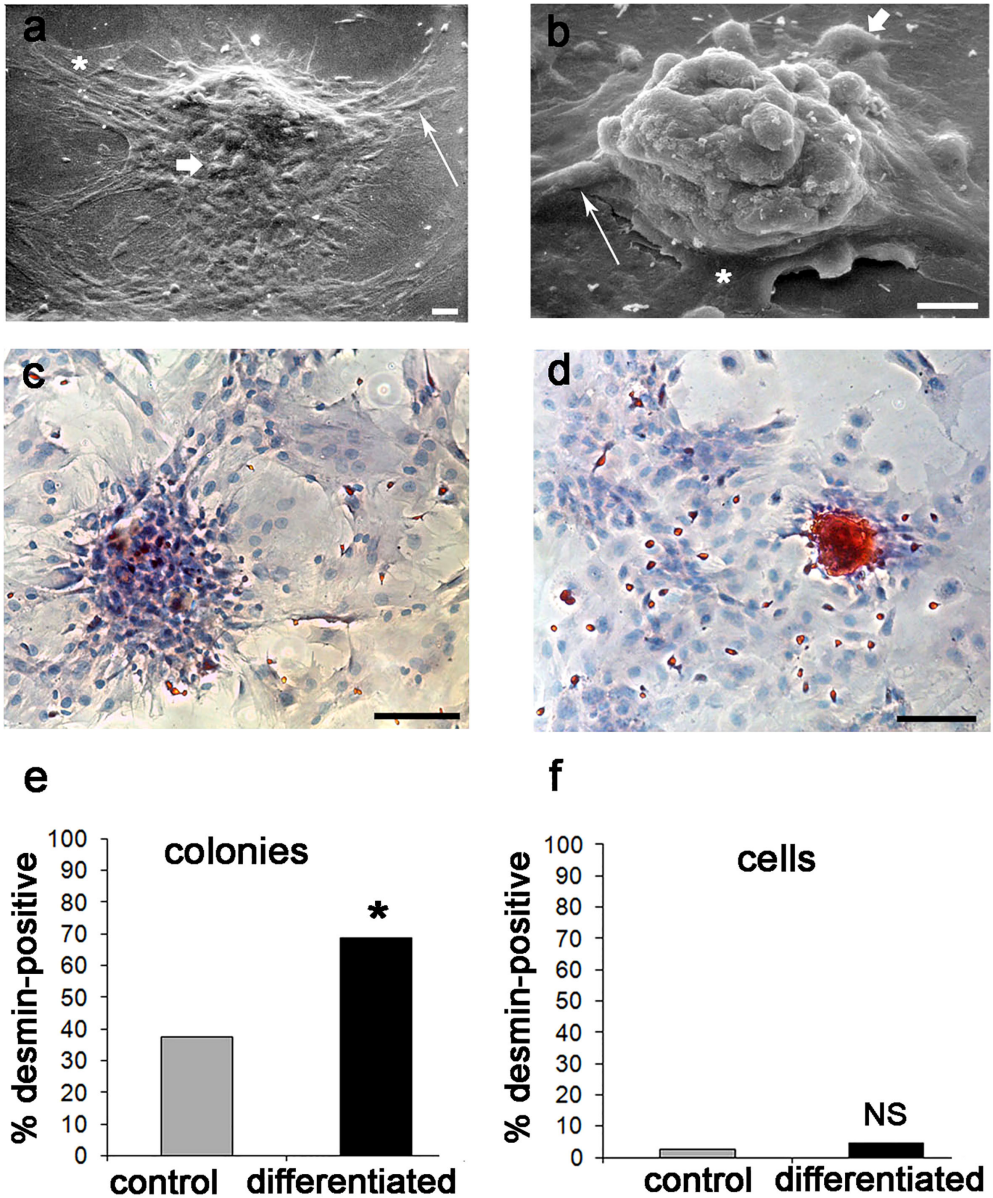
Ultrastructural morphology, DES immunostaining, and number of IR-DES D colonies, and single cells outside the colonies in control and differentiated rBMSCs grown on polystyrene plastic, at 21 days: **(A)** SEM of a control D colony; **(B)** SEM of a differentiated D colony. Note that all cell morphotypes are contributing to the colonies, including fibroblast-like (long arrow), polygonal (short arrow), and large-flattened (asterisk) ones. However, there is a substantial change in colony morphology between control and differentiated ones, with osteogenic colonies being more packed and humped with respect to controls; **(C)** LM of IR-DES in control D colony; **(D)** LM of IR-DES in differentiated D colony. Note increased intensity and extent of IR-DES in the differentiated colony with respect to the control one; **(E)** percent distribution of IR-DES D colonies, in control and differentiated cultures. Note a statistically significant increase in the proportion (here expressed as a percentage) of IR-DES D colonies in differentiated versus control cultures; **(F)** percent distribution of IR-DES cells growing outside the colonies, in control and differentiated cultures. No statistically significant difference in cell proportions (here expressed as percentages) was observed. Each SEM image represents the result of a sample selected from an average of three different experiments, whereas LM images are taken from the same index culture. Each graph represents the result of the same index experiment (courtesy of Dr. Elena Bassi, Feliciani-Ferretti Fund 2013 –2014 and post-doctoral Fellowhips 2014 –2016, UNIPR, Parma, Italy). Bars **(A)** = 20 microns; **(B)** = 10 microns; **(C, D)** = 100 microns; NS, not significant; * = p *< 0.05.

Further confirmation of these results was achieved by SEM analysis. At 21 days, osteogenic differentiation induced packing and humping of D colonies (**Figure 8B**) with respect to controls (**Figure 8A**); however, all cell morphotypes were observed to contribute to colony formation both in control and differentiated colonies, as anticipated by LM studies (see above). In addition, in D colonies osteogenic induction increased the staining intensity for DES (**Figure 8D**) with respect to controls (**Figure 8C**). This qualitative evidence was supported by an increase of around three times in the colony-forming efficiency (CFE) of IR-DES D colonies between control (CFE = 4.4) and differentiated (CFE = 11.1) cultures, and a statistically significant increase in the proportion of IR-DES D colonies in differentiated with respect to control cultures (**Figure 8E**). In contrast, the proportion of IR-DES cells outside the colonies between controls and differentiated cultures remained constant (**Figure 8F**). Therefore, DES expression resulted as a direct reflection of the skeletogenic process by rBMSCs and their selfassembly in bone nodules.

**Figure 9:**
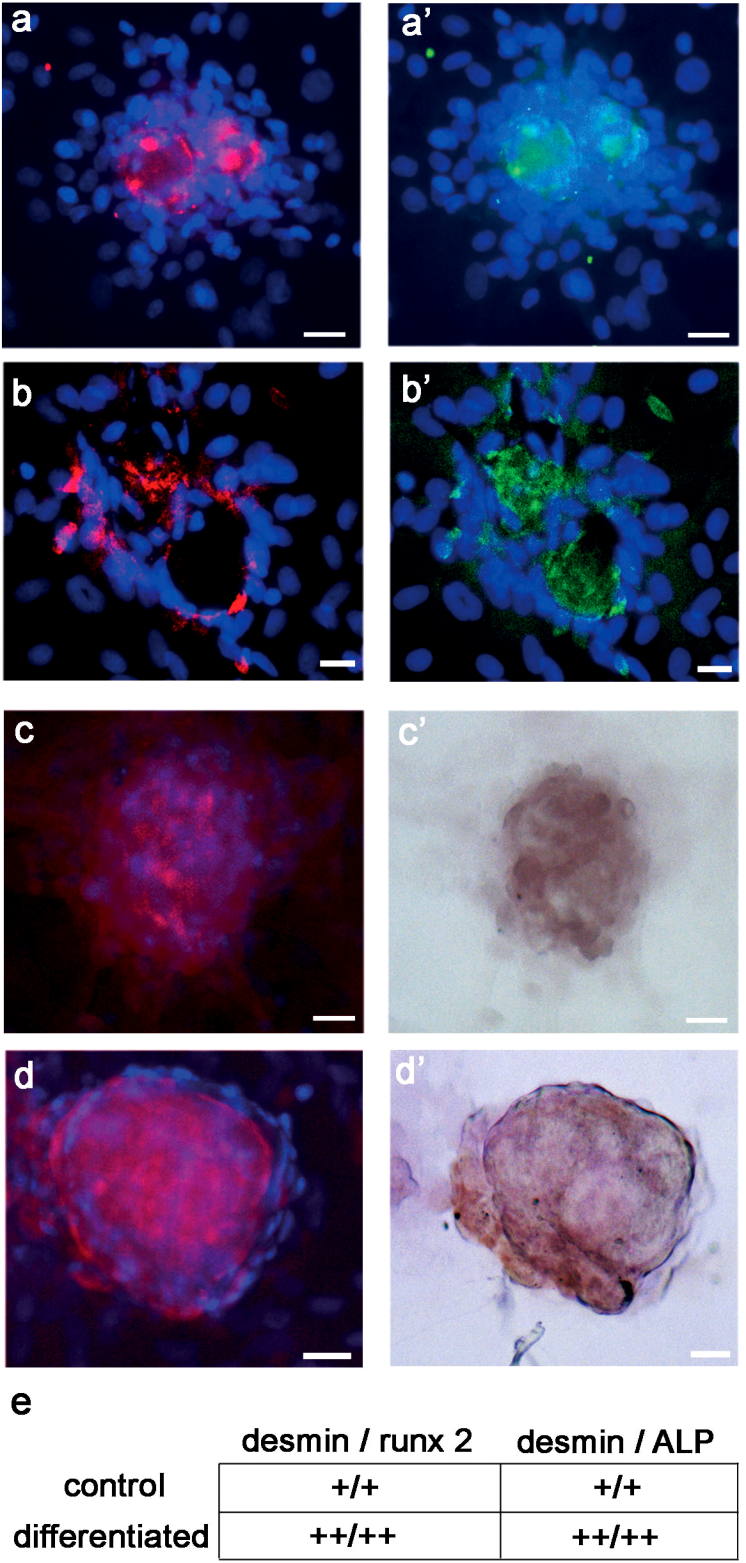
LM double-labeling IF, and combined IF/IC of DES (red color), runx2 (green color), and ALP (brown color) in control and differentiated D colonies, grown on polystyrene plastic for 21 days: **(A - A’)** control D colony, showing co-localization of **(A)** DES and **(A’)** runx2; **(B - B’)** differentiated D colony, showing co-localization of **(B)** DES and **(B’)** runx2. Note the increase in staining extent for DES and runx2 in the differentiated with respect to the control colony; **(C - C’)** control D colony, showing co-localization of **(C)** DES and **(C’)** ALP; **(D - D’)** differentiated D colony, showing co-localization of **(D)** DES and **(D’)** ALP. Note the increase in staining extent for DES and ALP in the differentiated with respect to the control colony; **(E)** semiquantitative evaluation of the staining intensity for DES, runx2 and ALP in control versus differentiated D colonies. An increase in staining (see unbiased criteria in Materials and Methods) was recorded for all markers in differentiated with respect to control colonies. (Courtesy of Drs. Alessandra Zamparelli, Grant FIRB RBAP10MLK7_004 post-doctoral Fellowship 2010- 2015 and Elena Bassi, Feliciani-Ferretti Fund 2013 –2014 and post-doctoral Fellowships 2014 –2016, UNIPR, Parma, Italy). Bars = 20 microns.

Finally, to support the assumption that DES was related to the molecular chain inducing preosteoblast and osteoblast differentiation, we searched for changes in its co-localization with runx2 and ALP. At 21 days, both runx2 and ALP co-localized with DES in a subpopulation of cells of the D colonies. Consistently, osteogenic differentiation increased the staining pattern of co-localized IR-DES/IR-runx2 (**Figures 9B-B’**) and IR-DES/IR-ALP (**Figures 9D-D’**) with respect to control cultures (**Figures 9A-A’, 9C-C’**). The semiquantitative analysis confirmed the LM staining features, indicating that immunolabeling intensity for DES, runx2, and ALP increased in the same differentiated cultures with respect to control ones (**Figure 9E**). These results corroborated all the previous evidence, substantiating the hypothesis that DES is a part of the molecular machinery activating master regulators of osteogenesis during the differentiation of rBMSCs.

## Discussion

In this study, we focused on the role of the cytoskeletal filament desmin (DES) in the *in vitro* osteogenic fate of bone marrow-derived mesenchymal stromal cells (BMSCs) from an experimental animal model, the adult male rat. Like their human counterpart, rBMSCs have intrinsic immunomodulatory properties (38), exhibit a stable immunophenotype and elevated multilineage differentiation potential at early culture passages (39), have similar morphotypes and replication features (40), and give rise to disordered and not remodeled woven bone (41), to some extent recapitulating the disordered and weakened osteoid observed in non-union fractures (NU), fibrous dysplasia (FD), and Paget disease of bone (Paget) (4–6). Collectively, rBMSCs can be exploited as a cellular model for *in vitro* mimicking the skeletogenic commitment of human BMSCs, and aspects of woven bone observable in the course of skeletal disorders like NU, FD, and Paget.

The immunophenotype characterization of our cells was based on the presence of the CD73 and CD90 antigens, a panel of markers less restrictive than those currently applied to human MSCs, on the assumption that in non-human species the antigenic immunoprofile is partly different but maintains constant expression of these two proteins (39). In addition, it conformed to the quantitative oscillations reported in male rats of the same strain, age, and at the same subculture passages (P2 - P4), and was identical to that of rBMSCs exhibiting a multipotential fate (20, 39, 43, 59). Consistently, contamination by the hematopoietic CD45 lineage was negligible. Similarly, as previously described (20) three well-characterized cell morphotypes were recognized, partly resembling phenotypes detected in human MSCs (60, 61), thus confirming that the procedure for rBMSCs isolation and expansion in monolayer was reliable. Finally, the growth of our cells on glass and polystyrene plastic was chosen based on the knowledge that rigid substrates favor the skeletogenic commitment of rBMSCs through the cytoskeletal machinery (62–64), thus enhancing the contribution of the mechanochemical transduction to the osteogenic differentiation.

### Desmin and the cytoskeletal machinery involved in the skeletogenic commitment of rBMSCs

All our rBMSCs contained a rich network of DES, whose molecular authenticity was confirmed by Westen blotting. The type III, intermediate filament (IF) DES (65) plays a well-recognized function in regulating structural and mechanical integrity, contractility, metabolism, and mitochondrial activity of skeletal muscle cells (66). However, it has also been found in human and rodent BM progenitors of the mesenchymal and hematopoietic lineages (10–24, 28, 30–32) exhibiting skeletogenic properties (25, 30, 32), in some instances accompanied by an increase in DES expression (29). Thus, DES emerges as a molecular partner in the array of signals leading to osteogenesis by BMSCs.

In our cell model, this hypothesis was initially substantiated by the evidence that DES co-localized with the two cytoskeletal proteins vimentin and F-actin, and the osteogenic transcription factor runx2. DES, vimentin, actin, and runx2 all exhibited an intracellular topography that varied in relation to the cell morphotype, with patterns of immunocytochemical staining reciprocally conforming to changes in the cell geometry, a property described for changes from elongated, to polygonal, to spread cytoplasms and related organelles arrangements, as a result of reciprocal reassembly of DES, vimentin, and actin filaments (67–69). Since this reassembly has been shown relevant to bring about runx2 expression (62, 70–73), we reasoned that knowledge of the cytoskeletal proteins and related enzymes/chaperones/signaling molecules detectable in our rBMSCs could be of utility to collect a panel of molecular information suitable for the eventual construction of a protein -protein interaction (PPI) network or interactome (74), focused on the potential role of DES in osteogenesis. Bioinformatic topological analysis of PPI networks, in fact, allows for robust predictions on biosignature and regulatory pathways of specific cellular processes (75).

To this purpose, we conducted a qualitative mass spectrometry (MS) analysis focussed on the cytoskeletal machinery including intermediate filaments, microfilaments, microtubules, contractile proteins, chaperones, and related enzymes/signaling molecules (76). Based on gene ontology clustering, we retrieved 85 proteins of which 43 were involved in the cytoskeletal structure, 36 contributed to its signaling and metabolic systems, and 6 acted as chaperones. Of these, we selected 29 proteins known as mechanochemical interactors in osteogenesis and immunomodulation by rodent osteoprogenitors comprising vimentin, actins, calumenin, transgelin-2, annexin-2, prohibitin, galectin-1, profilin-1, cofilin-1, talin-1, moesin, tubulins, vinculin, thymosin beta10, prelamin - A/C, myosins, troponins, tropomyosins, heat shock protein beta-1 or Hsp27, Hsp10, Hsp60, Hsp70, Hsp90, and zyxin (45–48, 77). Similarly, signaling molecules included the Rho GDP-dissociation inhibitor 1, cytoplasmic aspartate aminotransferase, calmodulin-2, myc box-dependent-interacting protein 1, and proteins S-100 (47, 48, 78, 79). Remarkably, MS detection levels (expressed as Log 2) of proteins in this molecular panel were in the interval of those recently reported for the entire proteome of rBMSCs (80), confirming the reliability of our analytical data for the construction of an interactome linking DES to the skeletogenic commitment of rBMSCs.

### The desmin interactome relevant to skeletogenic commitment of rBMSCs

Based on the above-mentioned cytoskeletal panel enriched with the two osteogenic regulators runx2 and alkaline phosphatase (ALP), and using a heuristic of sequential interactomics simulations to obtain an uninterrupted chain of connections, we constructed a PPI network whose robustness was supported by 10 predicted functional partners, automatically selected by the STRING algorithm at a very high level of confidence (score 0.9), that secured the reliability of the informational pathway (81).

The STRING bioinformatic resource is an *in silico* platform combining knowledge of structural and/or functional protein interactions, based on published experimental evidence, protein grouping in relation to metabolic, signaling, and/or transcriptional pathways, and *de novo* expected protein-protein associations based on genomic information. All these associations are also extended beyond the organism of their original description, by automatic transfer to orthologous protein pairs throughout the phylogenetic tree. In addition, the STRING reference database for genes and genomes is the internationally-recognized KEGG (Kyoto Encyclopedia of Genes and Genomes), which provides the system with one of the highest data accuracy and knowledge coverage available. Thus, STRING offers a very robust level of reliability in the proposed protein chains, ensuring also evolutionary conservation of the predicted interactions. Consistently, all sources of protein interactions included in our desmin interactome were made available in the relative figure legend. In the case of our analysis, a further level of strengthening was introduced, represented by a computational analysis of topologically-relevant indices of the PPI network, as resulting from the STRING graph. In this manner, we scaled down the set of potential protein interactions to a subset of the most likely functional events, further increasing the level of accuracy of the informational protein flow (82).

In particular, the topological analysis revealed that the molecule immediately downstream of the Rho GDP-dissociation inhibitor 1, i.e. the Ras homolog gene family member a (Rhoa) represented a primary site for trafficking of DES-related osteogenic interactions (top 10% highest degree k value), and a regulatory switch for information flowing throughout the network (top 10% highest between centrality value), being both a “hub” and a “bottleneck” of the PPI network (83, 84). As a result, Rhoa would collect inputs from genes critical for the progression to the osteogenic markers runx2 and ALP, and would itself be a gene essential to the predicted osteogenic pathway.

Rhoa is a small GTPase that, after activation of its associated kinase ROCK may induce the skeletogenic commitment of BMSCs in relation to a stiff growth environment, favoring assembly of the actin/actomyosin cytoskeleton, whose progressive rigidity favors osteoblastic differentiation through nuclear translocation of osteogenic transcription factors of the SMAD family. However, to complete osteoblastic maturation the Rhoa/ROCK system has to be inactivated (47). Since the Rhoa/ROCK/SMAD chain may impinge onto the runx2 transcription machinery (85), whereas cytoskeletal proteins downstream to DES like cofilin-1 and moesin contribute to restraining the Rhoa-dependent actomyosin assembly for BMSCs differentiation (47, 86), it is conceivable that the DES interactome converges to Rhoa as a primary regulatory node. This is also consistent with the evidence that in skeletogenic commitment of BMSCs, the Rhoa/ROCK signal machinery connects extracellular mechanosensory signals to the intracellular cytoskeleton, leading to nuclear translocation of the runx2 activators YAP/TAZ (87), whose cytoplasmic –nuclear shuttling requires anchoring of DES to the plasma membrane (88).

Similarly, topological indexes highlighted that moesin was another hub (top 10% highest degree k value) for trafficking of DES-related osteogenic interactions, whereas runx2 was another bottleneck (top 10% highest between centrality value) for osteogenic differentiation. According to the initial assumption of a preeminent cytoskeletal mechanism in DES-mediated osteogenesis, molecules like Rhoa, moesin, and runx2 provided the leading pathway or backbone of the interactome (83), ultimately directed to ALP. In contrast, DES resulted with lower k and closeness centrality values, and higher eccentricity values, indicating a role as an early signal of the global interactome. However, the overall information pathway resulted in robust interconnection (medium-high average clustering coefficient value), a feature typical of networks where proteins exert roles of reciprocal biological importance (49), corroborating the general assumption that DES was a molecular player in osteogenesis by BMSCs.

As an upstream effector of the interactome, DES established direct connections with vimentin, a feature observed primarily in muscle cells (66). In osteoblasts, vimentin can act as a negative regulator of osteogenesis (70), inhibiting osteoblast differentiation and reducing bone mass through direct transcriptional inhibition of the activating transcription factor 4, ending up with inhibition of osteocalcin and upstream ALP genes (89). Thus, restraint of vimentin inhibition by DES could serve to ensure the transition from immature to mature osteoblasts. A similar possibility is enforced by the evidence that DES may also interfere with the capacity of vimentin to sequester a large pool of intracellular collagen α1 mRNA, permitting its mobilization into the translational pathway during BMSCs osteogenic differentiation (90). As a result, collagen type I could be deposited in the bone extracellular matrix (ECM), to form the osteoid scaffold (91).

Remarkably, DES shares with vimentin also the capacity to bind O-linked N-acetylglucosamine (O-GlcNAc)-modified Ser/Thr residues of preosteoblast differentiation proteins, like runx2 (92). Since post-translational O-GlcNAc modification of runx2 increases its transcriptional activity and that of the runx2-target ALP (93), stabilization by DES of O-GlcNAcylations could favor upstream cytoskeletal-related signals governing runx2-dependent downstream transcriptional programs, like the ALP gene. In particular, progression from immature to mature osteoblasts implies functional ALP, that in turn requires downregulation of runx2 (85) through proteasomal degradation (94), as well as activation of the aerobic glycolytic metabolism (95).

Consistent with this evidence, myosins and actin interactors like cofilin-1, profilin-1, and moesin may associate with cytoplasmic proteasomal complexes (96) whereas DES, vimentin, and actin filaments are necessary for the functioning of the aerobic glycolytic metabolism in mitochondria (66, 97). The latter include the malate - aspartate shuttle and its enzymes malate dehydrogenase and aspartate aminotransferase, both contributing to the proposed interactome. Indeed, osteogenic differentiation of mouse BMSCs relies on the availability of aspartate aminotransferase-dependent glutamate (98), whereas inhibition of aerobic glycolysis by hypoxia leads to inhibition of ALP expression and mineralization in rat osteoblasts (99). Collectively, this interactomic evidence strongly supported a role for DES in the process of osteogenesis by rBMSCs.

### Desmin modifications in the course of rBMSCs osteogenic differentiation

In undifferentiated monolayer cultures, the growth of rBMSCs on stiff plates of polystyrene plastic and glass spontaneously led to colonies having two different cell densities, sparse (S) and dense (D). This phenomenon was previously reported to occur with human BMSCs grown on similar monolayers and interpreted as an index of variable differentiation potential of their constituent cells (100), likely representing multipotent stem cells and intermediate osteoprogenitors in active replication. In contrast, surrounding cells should be considered as not replicating and more differentiated (101). Remarkably, both rBMSCs colony types showed expression of DES in their cells, as expected by the evidence that all immunophenotypically-characterized morphotypes expressed DES. Therefore, supported by the bioinformatic evidence of a robust cytoskeletal chain linking DES to runx2 and ALP, we induced osteogenic differentiation of our rBMSCs, with the intent to explore whether DES, runx2, and ALP had congruent changes at the end of the differentiation period.

Being aware of the cell heterogeneity of our cultures, we used a classical differentiating cocktail based on β-glycerophosphate, ascorbic acid, and dexamethasone expected to recruit stem cell/osteoprogenitor populations with possibly different osteogenic responses (102). Consistently, we observed that all three cell morphotypes contributed to the colonies. In this system, S and D colonies were present in both control and differentiated cultures, though D colonies were predominant in both conditions, as observed in standard cultures of human BMSCs (100). Similarly, after 21 days of differentiation, only D colonies were more compact and more circular than their undifferentiated counterparts, a morphological feature related to the highest differentiation potential in human BMSCs (100). In rBMSCs, these geometrical rearrangements were connected to the capacity of mineral nodules to replete only the D colony, as expected by the tendency of differentiating colony-forming cells to give rise to multilayered three-dimensional “humps”, grossly recapitulating the organized lamellar/compact bone (91) where osteoblasts remain entrapped inside the mineralized woven bone as primitive osteocytes (103).

Mineralization of woven bone nodules proceeded with a contemporary striking increase in the number of IR DES D colonies, but not in the surrounding free cells of the culture. As a result of the strong packing of all cell morphotypes in D colonies, no clear distinction of the IR-DES cell morphotype/morphotypes involved was achieved. However, during mineralization, a parallel increase in IR-DES, IR-runx2, and IR-ALP occurred in a subpopulation of cells of the D colonies. This raises the possibility that some specific rBMSCs morphotypes might have a higher susceptibility to proceeding through the preosteoblast to the osteoblast phenotype, as we predicted in the recent past (20). Separation and isolation by density gradients of the various IR-DES cell morphotypes might eventually be used to clarify this point. Collectively, these observations corroborated the idea that DES was used by differentiating osteoprogenitors to trigger deposition of calcium in the ECM and was a regulator of downstream effectors in the osteogenic pathway of rBMSCs. Indeed, DES has been identified as a cytoskeletal controller of intracellular Ca^2+^: in non-excitable cells, DES blunts the Store-operated Ca^2+^ entry (SOCE) system by slowing diffusion of the stromal interaction molecule 1 (STIM1) to the plasma membrane. It follows reduced activation of the ORA1 channel and related intracellular entry of Ca^2+^, preventing excess Ca^2+^ inflow and cellular stress (37). In contrast, alterations in the DES - STIM1 complex might contribute to the pathogenesis of tubular aggregate myopathy (TAM) (37). TAM shares with Stormoken syndrome (STRMK) gain-of-function mutations of STIM1 and ORAI1 (104–106), leading to excess intracellular calcium influx, possibly favored by a reduced DES action on the SOCE pathway.

In corresponding targeted transgenic mice (33, 34), these mutations are associated with an altered bone architecture including reduced body size and short stature, reduced number of ribs and deformities of the vertebral column, osteoporotic-like lesions, decreased bone mechanical properties, increased trabecular/cortical bone fraction, bones thinner and more compact than in control animals, and reduced BM cavity. Consistently, TAM and STRMK patients have been anecdotally described with bone dysplasias including short stature, pectus excavatum, and saddle nose (35, 36). Therefore, DES emerges as a regulatory signal in the molecular pathway linking pre-osteoblast/immature osteoblasts to mature osteoblasts, the formation of woven bone, and mineralization.

Finally, through a transmembrane domain, DES might further influence the rate of calcium deposition in the osteoid. Indeed, DES can bind GlcNAc glycosaminoglycans (GAGs) and related *O*-GlcNAc GAGs (107), whose hyaluronic acid and heparan sulfate are the more abundantly released by differentiating BMSCs (108). Since GlcNAcGAGs foster osteogenesis by inhibiting the sclerostin - LRP 5/6 Wnt signaling pathway (109) and stabilizing the osteoprotegerin binding to RANKL (110), anchorage of DES to ECM GlcNAcGAGs could prevent bone resorption by osteoclasts, as recently observed when hyaluronic acid is anchored to the growth surface of co-cultured, murine osteoblasts and osteoclasts (111).

### Potential role of desmin in the early diagnosis and follow-up of non-union fracture healing, and endocrine -metabolic bone disorders

Increased expression of DES at early phases of the skeletogenic commitment of BMSCs might offer a new glimpse into altered woven bone metabolism and mineralization as occurring in the course of NU, FD, and Paget. A common clinical feature of these skeletal disorders is the delay in diagnosis and difficulties in follow-up of lesions (1–3). Indeed, current blood markers of bone remodeling have a limited application, especially in cases of low bone turnover where the formation of irregular, weakened and hypomineralized woven bone cannot be predicted and/or adequately monitored (4–6). All these conditions are characterized by derangement of osteogenesis by BMSCs (7–9). Since DES emerges as an initial signal in the cytoskeletal machinery activated by BMSCs during their skeletogenic commitment, we raise the possibility that highly sensitive, specific, and accurate analytical technologies could be exploited to identify circulating and/or tissue levels of DES in these disorders. Examples of this type of measurement include levels of blood/tissue DES by mass spectrometry in the course of human heart failure (112, 113), and blood vimentin in stroke, using the multiplexing Luminex protein platform (114). Primarily in NU, we predict that the absence/reduced increase in blood/tissue DES with respect to regular healing in bone fractures would readily suggest hampered BMSCs osteogenesis, favoring adequate therapeutic intervention. In contrast, increased blood/tissue levels of DES with respect to normal control subjects could be expected during subcutaneous osteogenesis in progressive osseous heteroplasia (115). This, is a result of inactivating mutations in the GNAS gene in skin MSCs, leading to reduced AMPc and increased self-assembly of DES filaments (116). The opposite would be plausible in the presence of inefficient osteogenesis in somatic activating mutations of the GNAS gene in FD (5), and disordered differentiation of BMSCs in Paget (9). In summary, detection and measurement of circulating and/or tissue DES might offer a new molecular tool for the clinical evaluation of osteoblast differentiation, woven bone formation, and binding of calcium to the ECM during altered skeletogenesis.

## Conclusions

The search for still unrecognized players of osteoblast differentiation, woven bone formation, and mineralization of the osteoid during BMSCs skeletogenesis has revealed DES as an upstream signal of a molecular pathway converging onto the bone master regulators runx2 and ALP, and its increased expression during bone nodules assembly. We believe this may open a new perspective for early diagnosis and follow-up of altered osteoblast metabolism, weakened woven bone, and insufficient mineralization by dysfunctional BMSCs as occurring in a number of skeletal disorders including NU, FD, and Paget.

## Data availability statement

The datasets presented in this study can be found in the repositories of the Laboratory of Regenerative Morphology and Bioartificial Structures - Re.Mo.Bio.S Lab of the DIMEC-UNIPR, Italy, and Centro Misure "Casnati" - UNIPR, Italy. They are available upon motivated request.

## Ethics statement

The animal study was approved by Ministry of Health, Italy, and UNIPR ethical commission. The study was conducted in accordance with the local legislation and institutional requirements.

## Author contributions

GC and FB supported and organized all the experimental activities developed at the Re.Mo.BIO.S lab - UNIPR and IRCCS Rizzoli Institue of Bologna by the postdoctoral research fellows mentioned in the text and engaged in the different sections of the project, collected and activated the software procedures for the interactomic analysis of the proteomic data, contributed to the various statistical analyses, and selected relevant studies of the international literature on topics detailed in the manuscript; NZ provided technical expertise for all light microscopic, immunocytochemical, and ultrastructural studies, and participated to the final review of the manuscript; GS developed the mathematical basis for cell culture morphometry, and supervised statistical analysis procedures; GR and LE guided, assisted, and adviced on the proteomic studies; SM, SC, MM contributed to evaluate the translational and clinical impact of the studies and their potential applications to humans; RT conceived all the experimental ideas and research design, leaded the research team, and wrote and critically reviewed the entire manuscript. All authors gave the final approval to the manuscript.
