# Supplementary material for: Woven bone formation and mineralization by rat mesenchymal stromal cells imply increased expression of the intermediate filament desmin
Source: Front Endocrinol (Lausanne). 2023 Sep 4;14:1234569. doi: 10.3389/fendo.2023.1234569 (PMC10507407; doi:10.3389/fendo.2023.1234569)
Supplement: Supplementary file 1 [file DataSheet_1.docx]

**Legends Supplementary Figures**

**Figure 1.** Distribution of cytoskeletal, chaperone, and related enzymatic interactors of rBMSCs as detected by qualitative mass spectrometry. Note the presence of tubulin, vinculin, heat shock protein beta-1 or Hsp27, Hsp60, Hsp70, Hsp90, and cytoplasmic aspartate aminotransferase. The role of each of these proteins was progressively included in the bioinformatic interactomic analysis of the DES-related osteogenic pathway, leading to partly interrupted chains of connections, with the exception of aspartate aminotransferase, which was retained in the final PPI network. Data were collected from two independent rBMSCs samples, and here shown the results from the sample with the best Log 2 values. (Courtesy of Drs. Elia Consolini, PhD Program in Molecular Medicine 2016-2019 and Andrea Faccini, “Centro Misure Giuseppe Casnati”, UNIPR, Parma, Italy).

**Figure 2**. Main steps for calculation of size and circularity, and their distribution in control and differentiated rBMSCs colonies, in the case of D colonies. The contour of control (a) and differentiated (b) colonies were highlighted in color, and transformed by edge detection (c, d). After appropriate numerical elaboration, the interval of values for planar size (e, f) and circularity (g, h) were graphed on a classical Cartesian frame. All values resulted in the homogeneous distribution along a continuum in all samples, suggesting that the variables were measured on homogeneous colony populations within a specific functional condition (i.e. control versus differentiated). These results confirmed the reliability and robustness of the methodology applied.
