# Supplementary material for: Woven bone formation and mineralization by rat mesenchymal stromal cells imply increased expression of the intermediate filament desmin
Source: Front Endocrinol (Lausanne). 2023 Sep 4;14:1234569. doi: 10.3389/fendo.2023.1234569 (PMC10507407; doi:10.3389/fendo.2023.1234569)
Supplement: Supplementary file 2 [file DataSheet_2.docx]

**Supplementary Materials and Methods**

***Animals, isolation of rat bone marrow-derived mesenchymal stromal cells (rBMSCs), and culture of C2C12 mouse myoblasts***

Initially, Sprague-Dawley male rats were deeply anesthetized with intraperitoneal (i.p) Zoletil (50 mg/kg), and euthanasia was reached by i.p. Penthotal Sodium (100 mg/kg) and cervical dislocation. Then, the femur BM was collected by inserting an 18-gauge needle into the bone diaphysis and, under sterile conditions flushing it into a sterile Petri dish with DMEM low glucose (LG) containing 10% fetal calf serum (FCS), and an antibiotic-antimycotic combination of 100 microg/ml penicillin, 10 mg/ml streptomycin (P/S), and 25 microg/ml amphotericin B (Euroclone, Italy). This solution was used as a culture medium. Then, BM mononuclear cells were isolated by gradient centrifugation on Histopaque-1077 (Sigma-Aldrich), washed twice in culture medium, counted in a Burker chamber, and plated on a standard plastic T25 flask (Corning, USA) at a density of 2 x 10^5^ / cm^2^. After 72 hs, non-adherent cells were removed, adherent cells re-seeded in a T25 (passage 0 or P0), and the medium was replaced every two to three days. At 80% confluence (i.e. subconfluence) cells were detached using trypsin 0.02% EDTA for 1 minute, split 1:2, re-seeded, cultured with the same procedure up to complete passage 4 or P4, and then used in the P2 - P4 interval for all experiments. Consistency in the morphology of rBMSCs in the culture interval used was checked using an inverted, phase-contrast light microscope (Zeiss Observer A1).

***Flow cytometric immunophenotyping*** ***of rBMSCs***

rBMSCs at P2 were incubated at 4°C for 15 min with monoclonal antibodies against rat CD45, CD73, CD90 (BD Pharmingen, USA), and their isotype controls (IgG2a, Abcam, UK) at a concentration of 1:20. Then, samples were washed with buffer, centrifuged, resuspended in the same buffer, and incubated with FITC-labelled secondary antibody (1:20, Dako, USA) for 15 min in the dark at room temperature (RT). After a final wash, cells were analyzed using a Becton Dickinson FACSCanto II, equipped with a blue laser capable of detecting light scattering. The FITC fluorescence was filtered by a 530 ± 21 nm bandpass filter. A single gate was applied on the forward versus side scatter, to allow selection of the suitable cell population. The frequency of positive cells was measured as the percentile of cells gated in the FITC channel with activities above 99.0% of the corresponding isotype control. Data were analyzed with the FACSDIVA software (Becton Dickinson).

***Light microscopic immunofluorescence, immunocytochemistry, and histochemistry of rBMSCs***

For light microscopic (LM) single-labeling immunofluorescence (IF), rBMSCs at P2 were grown on 1.13 cm^2^ glass coverslips at a density of 2 x 10^4^/cm^2^ for 7–14 days, and fixed with 70% cold ethanol at 4C° for 30 min. After washing with PBS (0.1 M, pH 7.4), the cells were incubated for 30 min in PBS containing 1% bovine serum albumin at RT, to block unspecific binding. Finally, cells were immunostained using either a polyclonal rabbit anti-DES (1:50, Abcam, UK) for 1h at RT, or a monoclonal mouse anti-vimentin (1:500, clone VIM 13., SIGMA, USA), or a rat anti-human RUNX2/CBFA1 to the isoform 2 (1:30, R&D Systems, USA) cross-reacting with rat, overnight at 4 C°. Then samples were washed in buffer, and incubated with either an anti-rabbit tetramethylrhodamine isothiocyanate (TRITC)-conjugated or a rabbit anti-mouse fluorescein isothiocyanate (FITC)-conjugated (cross-reacting with rat) secondary antibodies (1:100, Dako, USA) for 1 h at RT.

For LM double-labeling IF, rBMSCs grown on glass or on 6-well plastic dishes (Corning, USA) were initially immunostained for DES (see above). In a second step, after repeated washes in PBS cells were immunolabelled using monoclonal antibodies, to either the rat CD45 (1:10), CD73 (1:10), or CD90 (1:50) (BD Pharmingen, USA) antigens, for 1 h at RT. All these antigens were visualized using a rabbit anti-mouse FITC-conjugated secondary antibody (1:100, Dako, USA) incubated for 1 h at RT.

For LM single-labeling immunocytochemistry (IC), control and differentiated rBMSCs grown on plastic were incubated with rabbit polyclonal anti-DES (1:50) for 72 hs at 4°C, as above. After rinsing in PBS, cells were treated with anti-mouse/anti-rabbit biotinylated IgG (1:200, Vectastain, Vector Laboratories, CA, USA) for 2 hrs at RT, and DES revealed with the avidin-peroxidase complex (ABC Kit Vectastain) reacting for 30 min at RT, followed by diaminobenzidine (DAB) as a chromogen. Cell nuclei were counterstained with Mayers hematoxylin (Sigma, St Louis, MO, USA) for 3 min, cells dehydrated in ethanols, and mounted with Eukitt (Sigma) for visual inspection.

For combined LM, double-labeling IF/IC control and differentiated rBMSCs grown on plastic (see above) were immunostained for DES (see single-labeling IF protocol), rinsed in PBS, and immunolabeled with a monoclonal anti-human bone and liver alkaline phosphatase (ALP) cross-reacting with rat (1:100, DSHB, The University of Iowa City, IA), for 72 hs at 4°C. After rinsing in PBS, cells were incubated with anti-mouse/anti-rabbit biotinylated IgG (1:200, Vectastain, Vector Laboratories, CA, USA) for 2 hrs at RT, and ALP revealed with the ABC Kit, (Vectastain) reacting for 30 min at RT, followed by DAB as a chromogen (see above).

For combined LM double-labeling histochemistry (HC) / IC, differentiated rBMSCs grown on plastic (see above) were initially stained for calcium deposition with alizarin red (see below osteogenic differentiation). Then, cells were rinsed several times in PBS to moderately bleach the weakly bound aqueous alizarin, and immunostained with rabbit polyclonal anti-DES (1:50) for 72 hs at 4°C and DAB as a chromogen, as detailed above (see LM single-labeling IC section). Cells were counterstained with Mayers hematoxylin (Sigma, St Louis, MO, USA) for 3 min, dehydrated in ethanols, and mounted with Eukitt (Sigma) for visual inspection.

For LM fluorescent HC, a 0.8mM stock solution in DMSO was prepared using TRITC-coupled phalloidin (P1951, Sigma). P2 rBMSCs grown on glass coverslips (see above) were fixed in 4% paraformaldehyde / PBS for 10 min at RT, rinsed in PBS, and permeabilized with 0.15% Triton X-100 for 7 min. Finally, cells were stained with TRITC-phalloidin diluted 1:50 in PBS, and incubated for 1h at RT.

Nuclei in all fluorescence preparations were counterstained and mounted with DAPI – DABCO. Monolayers were observed with either a Nikon Eclipse E600 or a Zeiss Axiophot. All images were captured using either a Nikon digital camera Dmx 1200 and NIS-Elements BR 2.20 software, or a Zeiss digital camera Axiocam MRc5, and Axiovision Rel 4.8 software.

***Scanning and transmission electron microscopy***

Three-dimensional (3D) morphology of rBMSCs and ensuing monolayer colonies grown on either plastic wells or glass coverslips at P2 (see below) were analyzed by scanning electron microscopy (SEM). Cells were fixed with 2.5% glutaraldehyde (GTA) in 0.1M sodium cacodylate buffer pH 7.4 for 1h at RT, dehydrated in graded series of ethanols, immersed in absolute acetone, and subjected to critical point drying. All samples were mounted on aluminum stubs and metalized for 90 s using gold sputtering, to cover cells with a 60 nm gold film. The preparations were examined using a Philips 501 apparatus, coupled with a Nikon Coolpix digital camera for the acquisition of images.

The subcellular organization of rBMSCs morphotypes was studied by transmission electron microscopy (TEM). Cell monolayers grown on glass coverslips at P2 (see below) were fixed with 2.5% GTA in 0.1M sodium cacodylate buffer, pH 7.4 for 1h at RT. Specimens were postfixed with 1% osmium tetroxide containing 1.5% potassium ferrocyanate (30 min at 4°C), and sequentially stained with 1% uranyl acetate (30 min at 4°C), and 1% tannic acid (Mallinckrodt, USA) in the dark for 30 min at RT. Samples were then dehydrated in graded series of ethanols and embedded in Epon 812 resin. The resin was then allowed to polymerize for 48 h at 60C°. The embedded monolayers were finally sectioned on an ultramicrotome (Ultracut E, Richert-Jung). Ultrathin sections (90 nm thick) were collected on 200-mesh nickel grids, stained with 3% uranyl acetate and Reynold’s lead citrate, and examined with a Zeiss EM109, TEM apparatus. Images were captured using a Nikon digital camera Dmx 1200F, and ACT-1 software.

***Western blot analysis of desmin in rBMSCs***

For Western blotting, rBMSCs were used at P2; C2C12 myoblasts were used as positive controls. The samples were homogenized in lysis buffer, and centrifuged at 5400 x g for 20 min at 4°C. The supernatant was collected, and the protein concentration was determined by spectrophotometry using the BCA protein assay kit (Pierce Biotech, USA). Sixty μg of the extracted proteins were denatured at 100°C for 5 min, loaded on a 10% SDS-PAGE, and after separation transferred to a nitrocellulose membrane. Overnight incubation was performed at 4°C using the anti-DES antibody (see above) diluted 1:200. Actin was immunoblotted using a polyclonal, goat anti-β-actin (1:2000 Santa Cruz Biotecnology) for 1h at RT, and used as a reference standard. The immunoreactive (IR) product was detected using horseradish peroxidize-conjugated, anti-rabbit, and anti-goat secondary antibodies, respectively (1:12000 Santa Cruz Biotechnology) for 1h at RT. Bands were revealed by a chemiluminescent substrate (ECL, Amersham), and semiquantitative analysis of the immunoblotted material was performed by densitometry (PowerLook 2100 XL, Biorad), using a Quantity One software (Biorad Lab. Inc.).

***Preparation of rBMSCs for mass spectrometry analysis***

rBMSCs grown in rat tail collagen were detached using trypsin 0.02% EDTA for 1 min, resuspended with 100 μl of 6M urea, centrifuged at 7840 x g for 5 min at 4°C, the supernatant collected, and proteins quantified in duplicate (Bradford kit/BCA kit). Buffer exchange was performed by adding 50mM ammonium bicarbonate to dilute urea until 2M, proteins were reduced with 5mM dithiotreitol (DTT) (Sigma Aldrich D0632) at 37°C for 55 min, alkylated with 10 mM iodoacetamide (IAA) (Sigma Aldrich 1149) 30 min in the dark at RT, and the reaction stopped with 5mM DTT at 37°C for 15 min. All samples were digested by incubation with 5μg/μl trypsin (Sigma Aldrich T4799) 1:25 enzyme/protein substrate ratio at 37 °C for 24 hrs, and the digestion stopped with 3 μL of 98% acetic acid. Then, a salt content reduction was performed using a C18 Supelco membrane, treated with 1 mL methanol, and washed with 1 mL of H2O + 1% trifluoracetic acid (TFA) solution. Following the sample load, a further wash with 500 μl of H2O + 0.1 % TFA solution, and a peptide elution with 1 mL of H2O/acetonitrile (40:60) mixture + 0.1 % TFA were applied. Finally, all samples were dried under nitrogen flow, and reconstituted in 100μl of H2O/acetonitrile (50:50) mixture + 0.1 % TFA.

***LC-LIT-Orbitrap XL qualitative mass spectrometry analysis of rBMSCs***

Tryptic separation of the digested proteins was carried out by using an XB C18 AERIS PEPTIDE (150 x 2.1 mm, 5μm) column (Phenomenex, Torrance, CA, USA) equipped with a pre-filtering column. Eluting mixtures included solvent A (0.1% aqueous formic acid, v/v) and B (0.05% formic acid, 100% acetonitrile, v/v) delivered under gradient elution at the flow rate of 200 μL/min. The gradient was set as follows: 0 min 2% solvent B, 4 min 2% solvent B, 150 min 90% solvent B, 155 min 90% solvent B, 170 min 2% solvent B. The mobile phase was delivered by a Dionex Ultimate 3000 chromatographic system (Dionex Corporation, San José, CA, USA) equipped with a 100-vial capacity sample tray. The volume of the injected samples was 5 μL. Qualitative protein analysis was obtained by mass spectrometry using an LTQ linear ion trap-Orbitrap XL instrument (ThermoScientific Corporation, San José, CA, USA), equipped with an ESI interface and controlled by Xcalibur software. Optimized conditions of the interface were as follows: ESI (ionization electrospray source) voltage 3 kV, capillary voltage 13 V, capillary temperature 275° C, tube lens 100 V, sheath gas flow, auxiliary gas flow, and sweep gas flow delivered at 40, 10, and 5 arbitrary units, respectively. In the first scan event (full scan), the m/z window was 200-2000 with a resolution of 30.000. The four highest m/z ratios over a threshold of 1000 counts were selected for collision-induced dissociation (CID) in the ion trap, with a normalized collision energy of 35 % in the collision cell.

***Data analysis, and bioinformatics***

Protein identification was made by the Andromeda search engine against the Rattus Norvegicus database (Uniprot proteome ID: UP000002494), containing 29,941 proteins, and the Homo Sapiens database (Uniprot proteome ID: UP000005640), consisting of 74,034 proteins. Trypsin was chosen as a specific digestion mode with up to two missed cleavages. Proteins N-terminal acetylation and oxidation of methionine were set as variable modifications and carbamidomethylation of cysteine as a fixed modification. The minimum peptide length was fixed at 7, and mass tolerances and false discovery rate (FDR) for both peptides and proteins were set at 1%.

***Osteogenic differentiation of rBMSCs***

rBMSCs at P2 (seeding density 2 x 10^4^/cm^2^) were grown on 6-well, plastic dishes (Corning, USA) until subconfluence, and then exposed to the osteogenic medium. For alizarin red staining, cultures were washed twice with PBS, and fixed with 10% buffered formalin for 1h at RT. After two additional washes in PBS, samples were dehydrated in graded series of ethanols, and stained for 10 min with 2 ml of an aqueous solution of 40 mM alizarin red (pH 4.2). To remove unbound staining, cells were rinsed twice with distilled water and examined with phase contrast, inverted light microscope (Zeiss Observer A1). Images were captured using a Zeiss digital camera Axiocam HRc10-33, and software AxioVision 4.

***Colony size and circularity of rBMSCs***

The image border of each colony analyzed was highlighted in color by two independent operators using a computer-aided graphic, and the limit of each colony was considered as the point where the density of cell nuclei was strikingly reduced while the cell cytoplasm enlarged with respect to the bulk of the other cells in the colony. In the first step, an edge detection of the image border was performed to yield a closed curve in the plane, based on a standard computational technique. Sampling of the border, then, was obtained using a uniform step equal to Π/100, for a total of 200 points representing the entire border. A pseudo-symmetry axis of the entire border was further computed in a way to give rise to a minimum and a maximum point with respect to a Cartesian system of reference, both points intercepting the closed border. As a result, the pseudo-symmetry axis was brought to coincide with the x-axis of the Cartesian system and the closed border was split into two numerical subsets, i.e. two curves, that were further numerically approximated by 2 functions. Using these functions, both the border length called "perimeter", and a shape area called "area" were obtained by integration, and expressed on a micrometer scale. In the final step, the circularity of the area was computed as 4 x Π x area/perimeter^2^, giving rise to a scale of 0 to 1, where 0 represented a non-circle shape and 1 was a perfect circle.
