# Supplementary figures and images for: Woven bone formation and mineralization by rat mesenchymal stromal cells imply increased expression of the intermediate filament desmin
Source: Front Endocrinol (Lausanne). 2023 Sep 4;14:1234569. doi: 10.3389/fendo.2023.1234569 (PMC10507407; doi:10.3389/fendo.2023.1234569)

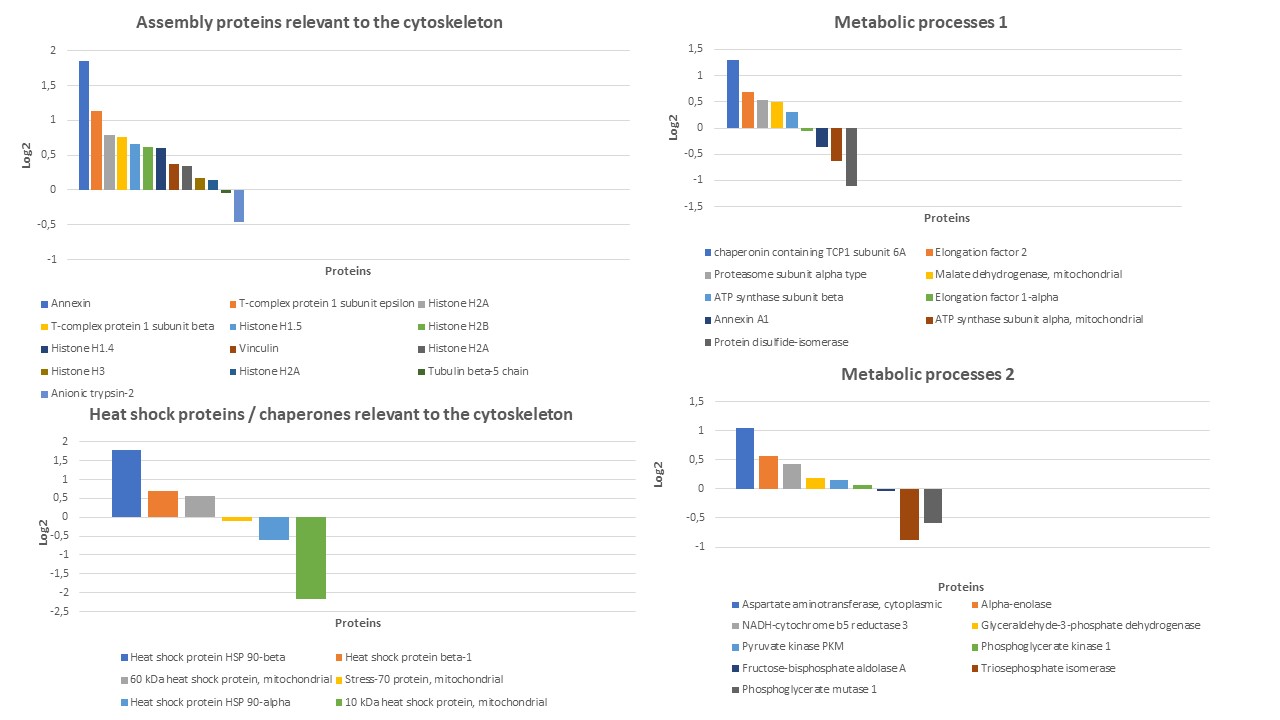

Supplement: Supplementary file 3 [file Image_1.jpeg]

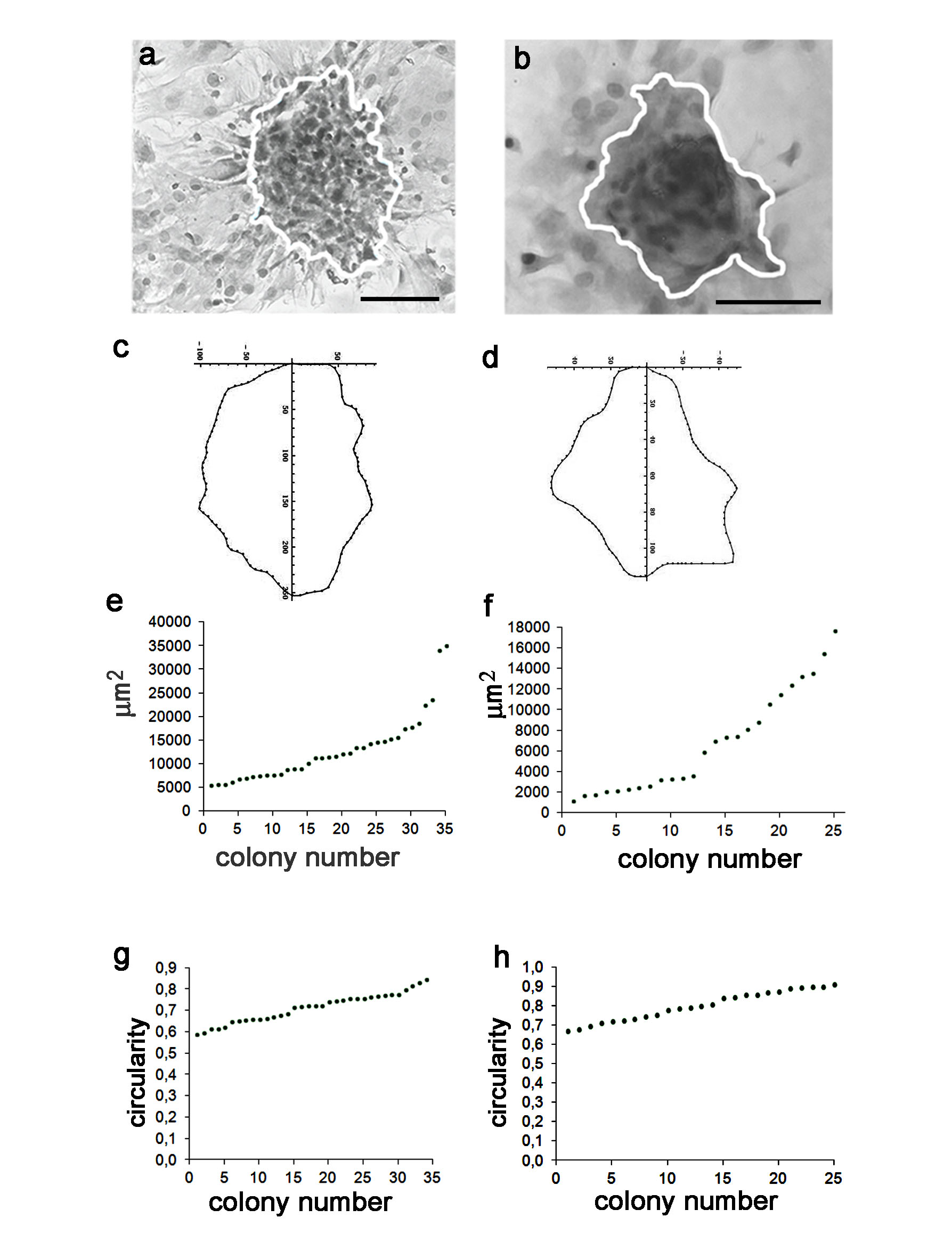

Supplement: Supplementary file 4 [file Image_2.jpeg]
